# Supplementary material for: First assessment of Iranian pomegranate germplasm using targeted metabolites and morphological traits to develop the core collection and modeling of the current and future spatial distribution under climate change conditions
Source: PLoS One. 2023 Feb 3;18(2):e0265977. doi: 10.1371/journal.pone.0265977 (PMC9897574; doi:10.1371/journal.pone.0265977)
Supplement: S1 Table — (DOCX) [file pone.0265977.s001.docx]

**S1 Table. Morphological traits and targeted metabolites of 152 pomegranate genotypes**

| Summary name | Origin | code | Number of fruits in tree | fruit mean weight | tree mean yield | Anthocyanin on Branch of This year | Petiole Color | Fruitful Flower Size | Fruit Albedo Color | Fruit Bottom shape | Fruit Heel shape | Juicy of Arils | Seed Size | Seed Color | Total Anthocyanins (mg/g) | Antioxidant Activity (%) | Total Polyphenols (mg/l) | Acidity (g/l) | Total Soluble Solid(%) | pH |
| --- | --- | --- | --- | --- | --- | --- | --- | --- | --- | --- | --- | --- | --- | --- | --- | --- | --- | --- | --- | --- |
| Jazireh | Bushehr | 1 | 7 | 3 | 1 | 3 | 1 | 5 | 2 | 3 | 2 | 3 | 3 | 3 | 16.3 | 27.3 | 1801.7 | 1.3 | 19.3 | 3.1 |
| Do-Mazeh | Chaharmahal and Bakhtiari | 2 | 7 | 3 | 3 | 3 | 1 | 5 | 2 | 4 | 1 | 3 | 3 | 6 | 7.1 | 48.2 | 1496.0 | 0.8 | 14.0 | 3.6 |
| Sefid-Rabi | Chaharmahal and Bakhtiari | 3 | 7 | 3 | 1 | 3 | 1 | 5 | 2 | 1 | 1 | 3 | 3 | 5 | 9.5 | 56.3 | 1988.2 | 1.2 | 15.3 | 2.8 |
| Sefid-Bakhtiari | Chaharmahal and Bakhtiari | 4 | 7 | 3 | 3 | 3 | 1 | 5 | 2 | 1 | 1 | 3 | 3 | 5 | 1.0 | 29.0 | 3531.0 | 0.7 | 14.2 | 3.3 |
| Khodroo | Chaharmahal and Bakhtiari | 5 | 3 | 3 | 1 | 3 | 1 | 5 | 2 | 4 | 1 | 3 | 3 | 6 | 21.5 | 37.3 | 1755.0 | 0.8 | 15.0 | 2.3 |
| Mesri | Fars | 6 | 7 | 3 | 3 | 3 | 1 | 5 | 2 | 3 | 3 | 3 | 3 | 1 | 36.0 | 46.2 | 1609.3 | 1.3 | 10.6 | 2.9 |
| Sabz-kazerun | Fars | 7 | 7 | 3 | 1 | 3 | 1 | 5 | 2 | 1 | 1 | 3 | 3 | 5 | 7.6 | 97.8 | 1911.0 | 1.7 | 11.2 | 2.2 |
| Farugh-Estahban | Fars | 8 | 7 | 3 | 3 | 3 | 1 | 5 | 2 | 1 | 1 | 3 | 3 | 5 | 51.3 | 49.6 | 1737.0 | 1.0 | 16.7 | 3.1 |
| Sabz-Sarvestan | Fars | 9 | 7 | 3 | 5 | 3 | 1 | 5 | 2 | 2 | 1 | 3 | 3 | 5 | 50.6 | 26.2 | 1139.0 | 1.0 | 16.8 | 2.9 |
| Shabestan-Poost-Koloft | Fars | 10 | 7 | 1 | 3 | 3 | 2 | 5 | 2 | 3 | 3 | 3 | 3 | 1 | 37.7 | 14.1 | 423.3 | 0.9 | 15.3 | 2.8 |
| Bihasteh-Jahrom | Fars | 11 | 7 | 3 | 3 | 3 | 1 | 5 | 2 | 1 | 1 | 3 | 3 | 5 | 2.8 | 21.2 | 755.0 | 0.8 | 14.9 | 3.1 |
| Bihasteh-Sarvestan | Fars | 12 | 7 | 3 | 3 | 3 | 3 | 5 | 2 | 1 | 1 | 3 | 3 | 5 | 7.6 | 26.8 | 496.0 | 0.5 | 13.6 | 2.5 |
| Atabaki-Sarvestan | Fars | 13 | 3 | 3 | 3 | 3 | 1 | 5 | 2 | 1 | 1 | 3 | 3 | 5 | 52.9 | 15.5 | 487.3 | 1.4 | 16.4 | 2.4 |
| Summary name | Origin | code | Number of fruits in tree | fruit mean weight | tree mean yield | Anthocyanin on Branch of This year | Petiole Color | Fruitful Flower Size | Fruit Albedo Color | Fruit Bottom shape | Fruit Heel shape | Juicy of Arils | Seed Size | Seed Color | Total Anthocyanins (mg/g) | Antioxidant Activity (%) | Total Polyphenols (mg/l) | Acidity (g/l) | Total Soluble Solid(%) | pH |
| Sabz-Ghasrdasht | Fars | 14 | 7 | 3 | 3 | 3 | 1 | 5 | 2 | 4 | 1 | 3 | 3 | 6 | 13.1 | 26.3 | 573.3 | 0.8 | 16.1 | 3.0 |
| Ghalatoon | Fars | 15 | 7 | 3 | 5 | 3 | 3 | 5 | 2 | 4 | 1 | 1 | 3 | 6 | 22.8 | 17.2 | 436.3 | 1.3 | 15.3 | 2.8 |
| Sefid-Seyedan | Fars | 16 | 7 | 3 | 3 | 3 | 2 | 5 | 2 | 2 | 1 | 3 | 5 | 2 | 16.2 | 25.3 | 396.7 | 1.0 | 14.6 | 2.2 |
| Berit-Ghermez | Fars | 17 | 7 | 3 | 5 | 3 | 2 | 5 | 2 | 2 | 1 | 3 | 3 | 2 | 53.6 | 27.6 | 589.0 | 1.1 | 16.1 | 3.2 |
| Kolukhi-Sarvestan | Fars | 18 | 7 | 3 | 5 | 3 | 1 | 5 | 2 | 1 | 1 | 3 | 3 | 2 | 9.0 | 49.8 | 1406.8 | 0.9 | 15.7 | 2.6 |
| Sakeli-Seyedan | Fars | 19 | 7 | 3 | 5 | 3 | 1 | 5 | 2 | 1 | 1 | 1 | 3 | 2 | 44.4 | 47.2 | 1931.0 | 1.4 | 16.0 | 3.7 |
| Tashtoo-Estahban | Fars | 20 | 7 | 3 | 3 | 3 | 1 | 5 | 2 | 1 | 2 | 3 | 3 | 4 | 19.1 | 44.1 | 1483.6 | 0.9 | 15.9 | 2.6 |
| Por-pea-Seyedan | Fars | 21 | 7 | 3 | 3 | 3 | 1 | 5 | 2 | 2 | 1 | 3 | 3 | 5 | 27.3 | 40.5 | 1095.5 | 0.9 | 15.2 | 3.2 |
| Oud-Neiriz | Fars | 22 | 3 | 3 | 1 | 3 | 2 | 5 | 2 | 4 | 1 | 3 | 3 | 6 | 9.7 | 64.6 | 2153.3 | 0.8 | 17.2 | 3.1 |
| Bazri-Neiriz | Fars | 23 | 3 | 3 | 1 | 3 | 2 | 3 | 2 | 3 | 3 | 1 | 3 | 1 | 8.3 | 41.9 | 1709.7 | 1.2 | 16.7 | 2.3 |
| Shabestan-Poost-Nazok | Fars | 24 | 7 | 3 | 3 | 3 | 1 | 5 | 2 | 4 | 1 | 3 | 3 | 6 | 49.0 | 42.2 | 1517.2 | 0.9 | 17.3 | 2.7 |
| Sefid-Estahban | Fars | 25 | 5 | 3 | 1 | 3 | 1 | 5 | 2 | 4 | 1 | 3 | 3 | 6 | 28.1 | 71.4 | 2370.3 | 0.9 | 16.0 | 3.0 |
| Summary name | Origin | code | Number of fruits in tree | fruit mean weight | tree mean yield | Anthocyanin on Branch of This year | Petiole Color | Fruitful Flower Size | Fruit Albedo Color | Fruit Bottom shape | Fruit Heel shape | Juicy of Arils | Seed Size | Seed Color | Total Anthocyanins (mg/g) | Antioxidant Activity (%) | Total Polyphenols (mg/l) | Acidity (g/l) | Total Soluble Solid(%) | pH |
| Robab-Torsh | Fars | 26 | 7 | 3 | 3 | 3 | 2 | 5 | 2 | 2 | 1 | 3 | 5 | 2 | 1.8 | 52.0 | 1318.7 | 0.8 | 13.3 | 2.3 |
| Narak-Marvdasht | Fars | 27 | 3 | 3 | 3 | 3 | 1 | 3 | 3 | 3 | 2 | 1 | 3 | 3 | 33.1 | 58.9 | 1886.5 | 1.0 | 15.4 | 2.2 |
| Kam-pea-Seyedan | Fars | 28 | 7 | 3 | 3 | 3 | 1 | 5 | 2 | 1 | 1 | 3 | 3 | 2 | 26.5 | 42.1 | 1393.0 | 0.9 | 16.2 | 3.1 |
| Gelo-Boland-Estahban | Fars | 29 | 7 | 3 | 5 | 3 | 1 | 5 | 2 | 1 | 2 | 3 | 3 | 4 | 43.7 | 52.0 | 1879.0 | 1.0 | 15.8 | 3.1 |
| Khajei-Ghasrdasht | Fars | 30 | 7 | 3 | 3 | 3 | 1 | 5 | 2 | 2 | 1 | 3 | 5 | 5 | 21.4 | 5.2 | 1269.7 | 0.9 | 15.1 | 3.2 |
| Shahvar-Ghsrdasht | Fars | 31 | 9 | 3 | 5 | 3 | 1 | 5 | 2 | 2 | 1 | 3 | 3 | 5 | 14.8 | 17.3 | 1209.2 | 0.5 | 14.6 | 3.4 |
| Narak-Sarvestan | Fars | 32 | 7 | 3 | 1 | 3 | 1 | 5 | 2 | 2 | 1 | 3 | 3 | 5 | 17.6 | 14.7 | 1529.7 | 1.0 | 15.5 | 2.4 |
| Atabaki-Sefid | Fars | 33 | 7 | 1 | 1 | 3 | 1 | 5 | 2 | 1 | 1 | 3 | 3 | 5 | 0.5 | 32.8 | 1514.0 | 0.7 | 17.8 | 3.2 |
| Robab-Sarvestan | Fars | 34 | 9 | 5 | 3 | 3 | 1 | 5 | 2 | 2 | 1 | 3 | 3 | 5 | 30.3 | 69.4 | 1947.0 | 0.7 | 16.6 | 3.2 |
| Bihaste-Neiriz | Fars | 35 | 3 | 3 | 1 | 3 | 1 | 5 | 2 | 2 | 1 | 3 | 3 | 5 | 34.8 | 45.4 | 2237.0 | 0.6 | 15.1 | 2.5 |
| Asali-Sarvestan | Fars | 36 | 9 | 3 | 5 | 3 | 1 | 5 | 2 | 2 | 1 | 3 | 3 | 5 | 24.9 | 42.2 | 2074.0 | 0.6 | 16.0 | 3.6 |
| Zard-poost-Koloft | Fars | 37 | 3 | 3 | 1 | 3 | 1 | 5 | 3 | 1 | 1 | 3 | 3 | 5 | 24.1 | 41.8 | 1689.6 | 1.0 | 13.2 | 3.2 |
| Robab-Sefid | Fars | 38 | 3 | 3 | 1 | 3 | 1 | 5 | 2 | 1 | 1 | 3 | 5 | 5 | 10.3 | 75.3 | 1827.5 | 1.5 | 9.8 | 3.2 |
| Summary name | Origin | code | Number of fruits in tree | fruit mean weight | tree mean yield | Anthocyanin on Branch of This year | Petiole Color | Fruitful Flower Size | Fruit Albedo Color | Fruit Bottom shape | Fruit Heel shape | Juicy of Arils | Seed Size | Seed Color | Total Anthocyanins (mg/g) | Antioxidant Activity (%) | Total Polyphenols (mg/l) | Acidity (g/l) | Total Soluble Solid(%) | pH |
| Farugh-Neiriz | Fars | 39 | 7 | 3 | 3 | 3 | 1 | 5 | 2 | 1 | 1 | 3 | 3 | 5 | 20.1 | 69.9 | 5321.8 | 1.1 | 11.1 | 3.3 |
| Shahvar-Marvdasht | Fars | 40 | 7 | 1 | 3 | 3 | 1 | 5 | 2 | 4 | 1 | 3 | 3 | 6 | 3.3 | 41.4 | 4723.9 | 0.6 | 13.7 | 3.4 |
| Berit-Sarvestan | Fars | 41 | 7 | 3 | 3 | 3 | 2 | 5 | 2 | 2 | 1 | 3 | 3 | 2 | 7.5 | 40.5 | 1531.7 | 0.9 | 11.9 | 3.3 |
| Khafari-Sefid | Fars | 42 | 7 | 3 | 5 | 3 | 1 | 5 | 2 | 4 | 1 | 3 | 3 | 6 | 12.9 | 53.2 | 4165.0 | 0.8 | 11.2 | 2.2 |
| Ramsar | Gilan | 43 | 7 | 3 | 3 | 3 | 1 | 5 | 2 | 2 | 1 | 3 | 3 | 2 | 46.2 | 53.5 | 1572.3 | 0.6 | 9.0 | 3.5 |
| Jangali-Rudbar | Gilan | 44 | 3 | 3 | 1 | 3 | 3 | 3 | 3 | 2 | 1 | 1 | 3 | 5 | 53.8 | 40.6 | 1687.0 | 0.8 | 16.1 | 2.8 |
| Kuhi-Rudbar | Gilan | 45 | 3 | 3 | 1 | 5 | 2 | 3 | 2 | 2 | 1 | 1 | 3 | 2 | 50.2 | 59.2 | 2068.3 | 0.7 | 16.1 | 2.7 |
| Sefid-Shahreza | Isfahan | 46 | 7 | 3 | 1 | 3 | 2 | 5 | 2 | 1 | 1 | 3 | 3 | 5 | 6.2 | 47.4 | 1526.3 | 0.8 | 15.5 | 3.2 |
| Sorkh-Malas-Shahreza | Isfahan | 47 | 7 | 5 | 5 | 3 | 3 | 5 | 2 | 2 | 1 | 3 | 3 | 2 | 48.5 | 26.7 | 1589.8 | 0.7 | 15.4 | 3.5 |
| Sorkh-Torsh-Shahreza | Isfahan | 48 | 7 | 3 | 1 | 3 | 1 | 5 | 2 | 3 | 3 | 3 | 3 | 1 | 44.6 | 27.0 | 1556.3 | 0.9 | 16.1 | 3.1 |
| Savehei-Dastjerd | Isfahan | 49 | 7 | 3 | 3 | 3 | 1 | 5 | 2 | 2 | 1 | 3 | 3 | 5 | 13.9 | 42.4 | 1854.7 | 1.2 | 15.3 | 2.9 |
| Bazri-Dastjerd | Isfahan | 50 | 3 | 3 | 1 | 3 | 1 | 3 | 3 | 1 | 1 | 1 | 3 | 2 | 29.6 | 48.2 | 1571.7 | 1.1 | 17.5 | 2.5 |
| Summary name | Origin | code | Number of fruits in tree | fruit mean weight | tree mean yield | Anthocyanin on Branch of This year | Petiole Color | Fruitful Flower Size | Fruit Albedo Color | Fruit Bottom shape | Fruit Heel shape | Juicy of Arils | Seed Size | Seed Color | Total Anthocyanins (mg/g) | Antioxidant Activity (%) | Total Polyphenols (mg/l) | Acidity (g/l) | Total Soluble Solid(%) | pH |
| Ardestani-Ghermez | Isfahan | 51 | 7 | 3 | 3 | 3 | 1 | 5 | 2 | 3 | 2 | 3 | 3 | 3 | 47.0 | 27.3 | 1603.7 | 0.9 | 15.2 | 3.0 |
| Abdandan | Isfahan | 52 | 3 | 1 | 3 | 3 | 1 | 5 | 2 | 2 | 1 | 3 | 3 | 5 | 44.3 | 31.3 | 1589.7 | 1.2 | 16.3 | 3.4 |
| Ghermez-Dastjerd | Isfahan | 53 | 7 | 3 | 3 | 3 | 1 | 5 | 2 | 2 | 1 | 3 | 3 | 5 | 12.5 | 43.7 | 1861.3 | 1.2 | 17.7 | 3.1 |
| Ghermez-Mamuli | Isfahan | 54 | 7 | 3 | 3 | 3 | 1 | 5 | 2 | 1 | 1 | 3 | 3 | 5 | 34.1 | 41.6 | 1717.0 | 1.0 | 16.3 | 2.8 |
| Keivani | Isfahan | 55 | 3 | 3 | 3 | 3 | 2 | 3 | 2 | 3 | 3 | 3 | 3 | 1 | 12.1 | 35.1 | 1145.0 | 1.2 | 15.0 | 2.4 |
| Yek-Kashan | Isfahan | 56 | 3 | 3 | 3 | 3 | 1 | 5 | 2 | 4 | 1 | 1 | 3 | 6 | 53.5 | 47.8 | 1521.5 | 0.6 | 18.4 | 2.7 |
| Binam-Dastjerd | Isfahan | 57 | 3 | 3 | 3 | 3 | 2 | 3 | 2 | 2 | 1 | 1 | 3 | 2 | 16.8 | 32.0 | 1365.9 | 0.8 | 15.1 | 3.0 |
| Naein | Isfahan | 58 | 3 | 1 | 3 | 3 | 1 | 5 | 2 | 2 | 1 | 3 | 3 | 5 | 17.4 | 22.2 | 1812.3 | 0.8 | 17.5 | 2.2 |
| Danehsiah-Dastjerd | Isfahan | 59 | 7 | 3 | 3 | 3 | 1 | 5 | 2 | 2 | 1 | 3 | 3 | 5 | 51.5 | 53.4 | 1683.7 | 0.9 | 16.0 | 3.0 |
| Homaabad | Isfahan | 60 | 7 | 3 | 3 | 3 | 2 | 5 | 2 | 3 | 3 | 3 | 3 | 1 | 5.3 | 79.0 | 5471.3 | 0.7 | 15.2 | 3.5 |
| Sefid-Babak | Kerman | 61 | 7 | 3 | 3 | 3 | 2 | 5 | 2 | 3 | 3 | 3 | 3 | 1 | 52.6 | 32.3 | 675.7 | 1.0 | 16.1 | 2.6 |
| Meikhosh-Babak | Kerman | 62 | 7 | 3 | 3 | 3 | 1 | 5 | 2 | 4 | 1 | 3 | 5 | 6 | 39.4 | 25.0 | 694.7 | 0.6 | 16.1 | 3.1 |
| Summary name | Origin | code | Number of fruits in tree | fruit mean weight | tree mean yield | Anthocyanin on Branch of This year | Petiole Color | Fruitful Flower Size | Fruit Albedo Color | Fruit Bottom shape | Fruit Heel shape | Juicy of Arils | Seed Size | Seed Color | Total Anthocyanins (mg/g) | Antioxidant Activity (%) | Total Polyphenols (mg/l) | Acidity (g/l) | Total Soluble Solid(%) | pH |
| Ghermez-Babak | Kerman | 63 | 7 | 3 | 3 | 3 | 1 | 5 | 2 | 1 | 1 | 3 | 3 | 5 | 43.1 | 25.7 | 1055.5 | 0.6 | 13.8 | 3.0 |
| Golabi-Bam | Kerman | 64 | 3 | 3 | 3 | 3 | 2 | 5 | 3 | 2 | 1 | 3 | 3 | 2 | 14.3 | 40.6 | 1485.3 | 0.8 | 16.6 | 2.9 |
| Mers-Bam | Kerman | 65 | 9 | 3 | 3 | 3 | 1 | 5 | 2 | 1 | 1 | 3 | 3 | 5 | 42.7 | 36.2 | 1132.5 | 0.6 | 18.0 | 2.2 |
| Sabi-Bam | Kerman | 66 | 3 | 3 | 3 | 3 | 3 | 3 | 3 | 4 | 1 | 1 | 3 | 6 | 1.8 | 74.5 | 1949.7 | 1.4 | 15.4 | 2.4 |
| Ghermez-Bam | Kerman | 67 | 3 | 3 | 3 | 3 | 2 | 5 | 2 | 2 | 1 | 3 | 3 | 2 | 4.7 | 59.1 | 7331.7 | 0.5 | 13.1 | 3.4 |
| Izeh | Khuzestan | 68 | 7 | 5 | 3 | 3 | 1 | 5 | 2 | 1 | 1 | 3 | 3 | 5 | 55.1 | 40.9 | 1785.7 | 0.8 | 16.9 | 2.8 |
| Danehsiah-Ramhormoz | Khuzestan | 69 | 7 | 3 | 3 | 5 | 1 | 5 | 2 | 3 | 2 | 3 | 5 | 3 | 52.2 | 44.0 | 1611.0 | 0.6 | 15.1 | 3.0 |
| Sinehpahn-Daneh-Ghermez | Khuzestan | 70 | 7 | 3 | 3 | 3 | 1 | 5 | 2 | 1 | 1 | 3 | 3 | 5 | 45.8 | 45.3 | 1658.0 | 0.9 | 17.8 | 2.8 |
| Bitalaf-Daneh-Sefid | Khuzestan | 71 | 7 | 3 | 3 | 3 | 1 | 5 | 2 | 1 | 2 | 3 | 3 | 4 | 12.9 | 49.8 | 1829.7 | 1.2 | 16.6 | 2.4 |
| Zaj-Ramhormoz | Khuzestan | 72 | 7 | 3 | 3 | 3 | 1 | 5 | 2 | 4 | 1 | 3 | 3 | 6 | 49.1 | 32.0 | 1394.5 | 1.2 | 15.6 | 3.1 |
| Sorkh-Daneh-Sefid | Khuzestan | 73 | 7 | 3 | 3 | 3 | 1 | 5 | 2 | 3 | 2 | 3 | 3 | 3 | 54.4 | 24.1 | 1736.2 | 0.8 | 17.8 | 2.3 |
| Yusefi-Dezful | Khuzestan | 74 | 3 | 3 | 3 | 3 | 2 | 5 | 2 | 3 | 3 | 3 | 3 | 1 | 46.8 | 47.7 | 2122.3 | 1.2 | 11.3 | 3.2 |
| Summary name | Origin | code | Number of fruits in tree | fruit mean weight | tree mean yield | Anthocyanin on Branch of This year | Petiole Color | Fruitful Flower Size | Fruit Albedo Color | Fruit Bottom shape | Fruit Heel shape | Juicy of Arils | Seed Size | Seed Color | Total Anthocyanins (mg/g) | Antioxidant Activity (%) | Total Polyphenols (mg/l) | Acidity (g/l) | Total Soluble Solid(%) | pH |
| Sefid-Boyer | Kohgiluyeh and Boyer-Ahmad | 75 | 7 | 3 | 3 | 3 | 1 | 5 | 2 | 2 | 1 | 3 | 3 | 5 | 48.3 | 7.2 | 1152.0 | 0.5 | 16.4 | 2.7 |
| Vahshi-Boyer | Kohgiluyeh and Boyer-Ahmad | 76 | 3 | 3 | 3 | 3 | 1 | 5 | 2 | 1 | 1 | 3 | 3 | 5 | 43.4 | 35.9 | 1453.5 | 0.6 | 14.7 | 2.2 |
| Sabz-Boyer | Kohgiluyeh and Boyer-Ahmad | 77 | 7 | 3 | 3 | 5 | 1 | 5 | 2 | 1 | 1 | 3 | 3 | 5 | 5.8 | 21.4 | 1531.0 | 0.5 | 17.2 | 3.2 |
| Mandali-Gorgan | Mazandaran | 78 | 7 | 3 | 3 | 3 | 1 | 5 | 2 | 1 | 2 | 3 | 5 | 4 | 52.5 | 36.6 | 1927.3 | 0.6 | 14.3 | 4.0 |
| Lamsari | Mazandaran | 79 | 3 | 3 | 3 | 3 | 1 | 5 | 2 | 3 | 3 | 1 | 3 | 1 | 18.3 | 18.6 | 1444.0 | 0.8 | 16.1 | 3.4 |
| Sisangan | Mazandaran | 80 | 7 | 3 | 3 | 3 | 1 | 5 | 2 | 1 | 1 | 3 | 3 | 5 | 46.4 | 33.3 | 1459.0 | 0.6 | 14.7 | 3.4 |
| Siahnar-Behshahr | Mazandaran | 81 | 3 | 3 | 3 | 3 | 1 | 5 | 2 | 2 | 1 | 3 | 3 | 2 | 10.9 | 50.3 | 1458.1 | 0.5 | 13.0 | 3.1 |
| Mohseni-Gorgan | Mazandaran | 82 | 7 | 3 | 3 | 3 | 1 | 5 | 2 | 1 | 1 | 3 | 5 | 2 | 14.8 | 19.4 | 1071.0 | 0.6 | 14.6 | 3.1 |
| Shekarnaz | Mazandaran | 83 | 7 | 3 | 3 | 3 | 1 | 5 | 2 | 3 | 3 | 3 | 3 | 1 | 22.5 | 27.7 | 1337.7 | 0.7 | 11.0 | 3.2 |
| Kabdar-Behshahr | Mazandaran | 84 | 7 | 3 | 3 | 3 | 1 | 5 | 2 | 1 | 2 | 3 | 5 | 4 | 34.3 | 52.7 | 1839.0 | 0.6 | 17.9 | 3.0 |
| Torsh-Behshahr | Mazandaran | 85 | 7 | 3 | 3 | 3 | 1 | 5 | 2 | 2 | 1 | 3 | 3 | 5 | 7.3 | 64.1 | 2479.7 | 1.5 | 13.8 | 2.4 |
| Summary name | Origin | code | Number of fruits in tree | fruit mean weight | tree mean yield | Anthocyanin on Branch of This year | Petiole Color | Fruitful Flower Size | Fruit Albedo Color | Fruit Bottom shape | Fruit Heel shape | Juicy of Arils | Seed Size | Seed Color | Total Anthocyanins (mg/g) | Antioxidant Activity (%) | Total Polyphenols (mg/l) | Acidity (g/l) | Total Soluble Solid(%) | pH |
| Babolsar | Mazandaran | 86 | 3 | 3 | 3 | 5 | 1 | 5 | 2 | 4 | 1 | 3 | 3 | 6 | 25.6 | 73.9 | 3492.3 | 1.8 | 16.9 | 2.2 |
| Ghermez-Gorgan | Mazandaran | 87 | 3 | 3 | 3 | 3 | 1 | 5 | 2 | 2 | 1 | 3 | 3 | 5 | 15.9 | 23.6 | 1118.0 | 0.7 | 14.5 | 2.8 |
| Savadkuh | Mazandaran | 88 | 7 | 3 | 3 | 3 | 1 | 5 | 2 | 1 | 1 | 3 | 3 | 2 | 6.8 | 58.0 | 2411.8 | 2.0 | 14.5 | 3.4 |
| Khoram-Deer-Gorgan | Mazandaran | 89 | 7 | 1 | 3 | 3 | 1 | 5 | 2 | 1 | 2 | 3 | 5 | 4 | 43.3 | 22.2 | 1396.3 | 0.7 | 16.1 | 3.0 |
| Zir-Ab | Mazandaran | 90 | 3 | 3 | 3 | 3 | 2 | 5 | 2 | 1 | 2 | 3 | 3 | 4 | 6.7 | 35.5 | 2154.3 | 2.2 | 16.7 | 2.5 |
| Shahi-Gorgan | Mazandaran | 91 | 7 | 3 | 3 | 5 | 1 | 5 | 2 | 4 | 1 | 3 | 3 | 6 | 51.7 | 22.9 | 1398.0 | 0.6 | 12.9 | 2.8 |
| Shirin-Behshahr | Mazandaran | 92 | 3 | 3 | 3 | 3 | 1 | 5 | 2 | 4 | 1 | 3 | 3 | 6 | 6.6 | 42.6 | 2183.7 | 0.9 | 17.1 | 3.3 |
| Sag-Nar-Lasjerd | Semnan | 93 | 3 | 3 | 3 | 5 | 1 | 5 | 2 | 1 | 1 | 1 | 3 | 2 | 40.7 | 16.0 | 1278.5 | 0.8 | 17.0 | 2.5 |
| Shahvar-Lasjerd | Semnan | 94 | 7 | 3 | 3 | 5 | 1 | 5 | 2 | 2 | 1 | 3 | 5 | 5 | 6.1 | 19.8 | 1224.7 | 0.6 | 16.0 | 3.3 |
| Porbar-Darjazin | Semnan | 95 | 7 | 3 | 3 | 3 | 1 | 5 | 2 | 1 | 1 | 3 | 3 | 5 | 29.3 | 57.0 | 1392.5 | 0.7 | 13.1 | 2.3 |
| Gharanchok-Lasjerd | Semnan | 96 | 7 | 3 | 3 | 3 | 2 | 5 | 2 | 2 | 1 | 3 | 3 | 5 | 4.0 | 47.8 | 1753.7 | 0.6 | 15.4 | 3.2 |
| Abdandan-Lasjerd | Semnan | 97 | 7 | 3 | 3 | 3 | 2 | 5 | 2 | 1 | 1 | 3 | 3 | 5 | 30.1 | 32.7 | 1744.3 | 0.8 | 17.5 | 3.0 |
| Narak-Lasjerd | Semnan | 98 | 3 | 3 | 3 | 3 | 1 | 5 | 2 | 3 | 2 | 3 | 3 | 3 | 8.1 | 20.4 | 1329.0 | 0.5 | 16.4 | 2.6 |
| Summary name | Origin | code | Number of fruits in tree | fruit mean weight | tree mean yield | Anthocyanin on Branch of This year | Petiole Color | Fruitful Flower Size | Fruit Albedo Color | Fruit Bottom shape | Fruit Heel shape | Juicy of Arils | Seed Size | Seed Color | Total Anthocyanins (mg/g) | Antioxidant Activity (%) | Total Polyphenols (mg/l) | Acidity (g/l) | Total Soluble Solid(%) | pH |
| Poost-Nazok-Darjazin | Semnan | 99 | 7 | 3 | 3 | 3 | 2 | 5 | 2 | 1 | 1 | 3 | 3 | 2 | 9.0 | 59.3 | 1226.3 | 0.6 | 9.1 | 3.5 |
| Ardestani-Darjazin | Semnan | 100 | 7 | 3 | 3 | 5 | 1 | 5 | 2 | 2 | 1 | 3 | 5 | 5 | 2.1 | 68.4 | 1418.3 | 1.4 | 12.6 | 3.3 |
| Shahvar-Sefid | Semnan | 101 | 7 | 3 | 3 | 3 | 1 | 5 | 2 | 2 | 1 | 3 | 3 | 5 | 0.8 | 25.1 | 1681.0 | 0.6 | 14.5 | 3.4 |
| Ardestani-Lasjerd | Semnan | 102 | 7 | 3 | 3 | 3 | 1 | 5 | 2 | 3 | 2 | 3 | 5 | 3 | 24.7 | 37.6 | 1342.3 | 0.8 | 15.1 | 3.0 |
| Mamuli-Lasjerd | Semnan | 103 | 3 | 3 | 3 | 3 | 1 | 5 | 2 | 1 | 2 | 3 | 3 | 4 | 41.1 | 24.8 | 1271.5 | 0.6 | 14.1 | 2.8 |
| Shahvar-Ghermez | Semnan | 104 | 7 | 3 | 3 | 3 | 1 | 5 | 2 | 3 | 3 | 3 | 5 | 1 | 36.4 | 17.9 | 1086.5 | 0.6 | 14.8 | 3.4 |
| Sarjou | Sistan and Baluchestan | 105 | 7 | 3 | 3 | 3 | 1 | 5 | 2 | 1 | 1 | 1 | 3 | 5 | 31.7 | 58.2 | 1692.7 | 1.3 | 17.4 | 3.2 |
| Ghermez-Bazmani | Sistan and Baluchestan | 106 | 7 | 3 | 3 | 3 | 1 | 5 | 2 | 2 | 1 | 3 | 3 | 5 | 52.0 | 31.2 | 1735.5 | 1.1 | 17.1 | 2.2 |
| Torsh-Vashik | Sistan and Baluchestan | 107 | 7 | 3 | 3 | 3 | 1 | 5 | 2 | 1 | 1 | 3 | 3 | 5 | 30.4 | 18.2 | 1072.5 | 0.8 | 15.2 | 2.9 |
| Bihasteh-Sangan | Sistan and Baluchestan | 108 | 9 | 7 | 3 | 3 | 2 | 5 | 2 | 3 | 3 | 3 | 3 | 1 | 2.2 | 8.4 | 428.7 | 0.6 | 12.5 | 3.1 |
| Yek-Kiluei | Sistan and Baluchestan | 109 | 7 | 1 | 3 | 3 | 2 | 5 | 2 | 3 | 3 | 3 | 3 | 1 | 35.3 | 35.2 | 740.5 | 1.2 | 15.9 | 3.1 |
| Sib-Hoshak | Sistan and Baluchestan | 110 | 9 | 7 | 3 | 3 | 3 | 5 | 2 | 4 | 1 | 3 | 3 | 6 | 4.8 | 24.6 | 434.7 | 1.2 | 14.5 | 2.5 |
| Meikhosh-Sangan | Sistan and Baluchestan | 111 | 7 | 1 | 3 | 3 | 2 | 5 | 2 | 2 | 1 | 3 | 3 | 2 | 27.5 | 13.5 | 367.0 | 1.1 | 15.3 | 3.3 |
| Summary name | Origin | code | Number of fruits in tree | fruit mean weight | tree mean yield | Anthocyanin on Branch of This year | Petiole Color | Fruitful Flower Size | Fruit Albedo Color | Fruit Bottom shape | Fruit Heel shape | Juicy of Arils | Seed Size | Seed Color | Total Anthocyanins (mg/g) | Antioxidant Activity (%) | Total Polyphenols (mg/l) | Acidity (g/l) | Total Soluble Solid(%) | pH |
| Golabi-Haste-Dorosht | Sistan and Baluchestan | 112 | 7 | 3 | 3 | 3 | 1 | 7 | 2 | 1 | 1 | 3 | 5 | 2 | 50.0 | 25.9 | 1541.3 | 0.9 | 16.5 | 3.2 |
| Bazmani-Poost-Nazok | Sistan and Baluchestan | 113 | 7 | 3 | 3 | 3 | 1 | 3 | 2 | 3 | 2 | 1 | 3 | 3 | 20.5 | 28.6 | 1730.3 | 1.1 | 15.4 | 2.5 |
| Malas-Sarjoo | Sistan and Baluchestan | 114 | 5 | 3 | 3 | 3 | 1 | 5 | 2 | 3 | 2 | 3 | 5 | 3 | 45.2 | 30.9 | 1526.4 | 1.3 | 16.2 | 3.3 |
| Mamuli-Zabol | Sistan and Baluchestan | 115 | 9 | 5 | 3 | 3 | 1 | 5 | 2 | 2 | 1 | 3 | 3 | 5 | 15.1 | 25.4 | 1181.3 | 1.1 | 16.5 | 2.1 |
| Savehei-Ghermez | Sistan and Baluchestan | 116 | 1 | 3 | 3 | 3 | 1 | 5 | 2 | 1 | 1 | 3 | 3 | 5 | 38.7 | 39.3 | 1618.3 | 0.9 | 15.8 | 3.1 |
| Nahug | Sistan and Baluchestan | 117 | 7 | 3 | 3 | 3 | 1 | 5 | 3 | 1 | 1 | 3 | 3 | 5 | 25.0 | 57.2 | 1995.7 | 1.3 | 15.8 | 3.1 |
| Golabi-Poost-Nazok | Sistan and Baluchestan | 118 | 7 | 3 | 3 | 3 | 2 | 5 | 2 | 3 | 3 | 3 | 3 | 1 | 36.2 | 30.1 | 1445.5 | 1.0 | 16.7 | 3.2 |
| Maamuli-Baluch | Sistan and Baluchestan | 119 | 7 | 3 | 3 | 3 | 2 | 5 | 2 | 2 | 1 | 3 | 3 | 2 | 15.2 | 40.6 | 1251.8 | 0.6 | 17.5 | 3.1 |
| Kuhak | Sistan and Baluchestan | 120 | 3 | 3 | 3 | 3 | 1 | 5 | 2 | 1 | 1 | 1 | 3 | 2 | 28.6 | 66.7 | 2151.8 | 1.0 | 14.8 | 2.7 |
| Nukjub | Sistan and Baluchestan | 121 | 3 | 3 | 3 | 3 | 1 | 5 | 2 | 3 | 2 | 3 | 3 | 3 | 53.2 | 33.0 | 1855.3 | 0.9 | 16.6 | 3.1 |
| Sefid-Bazmani | Sistan and Baluchestan | 122 | 3 | 1 | 3 | 3 | 1 | 5 | 2 | 3 | 2 | 3 | 3 | 3 | 49.9 | 36.6 | 1095.5 | 0.6 | 15.1 | 2.6 |
| Malisak-Hoshak | Sistan and Baluchestan | 123 | 9 | 9 | 3 | 3 | 1 | 9 | 2 | 1 | 2 | 3 | 3 | 4 | 16.9 | 49.7 | 2045.0 | 1.1 | 17.5 | 3.1 |
| Malas-Nukjub | Sistan and Baluchestan | 124 | 9 | 5 | 3 | 3 | 1 | 5 | 2 | 1 | 1 | 3 | 3 | 5 | 24.2 | 11.6 | 1164.0 | 1.4 | 15.9 | 2.8 |
| Summary name | Origin | code | Number of fruits in tree | fruit mean weight | tree mean yield | Anthocyanin on Branch of This year | Petiole Color | Fruitful Flower Size | Fruit Albedo Color | Fruit Bottom shape | Fruit Heel shape | Juicy of Arils | Seed Size | Seed Color | Total Anthocyanins (mg/g) | Antioxidant Activity (%) | Total Polyphenols (mg/l) | Acidity (g/l) | Total Soluble Solid(%) | pH |
| Bihaste-Ladiz | Sistan and Baluchestan | 125 | 9 | 5 | 3 | 3 | 2 | 5 | 2 | 3 | 3 | 3 | 3 | 1 | 41.8 | 66.4 | 3653.0 | 0.9 | 16.3 | 3.0 |
| Sefid-Bazmani | Sistan and Baluchestan | 126 | 3 | 3 | 3 | 3 | 2 | 5 | 2 | 3 | 3 | 3 | 3 | 1 | 3.6 | 61.7 | 3126.2 | 1.3 | 11.6 | 2.6 |
| Khorasani-Sefid | Sistan and Baluchestan | 127 | 7 | 3 | 3 | 3 | 1 | 5 | 2 | 4 | 1 | 3 | 3 | 6 | 19.6 | 64.7 | 2555.0 | 1.6 | 13.6 | 3.0 |
| Bazmani-Poost-Koloft | Sistan and Baluchestan | 128 | 5 | 3 | 3 | 3 | 2 | 5 | 2 | 2 | 1 | 3 | 3 | 2 | 1.9 | 58.9 | 5060.4 | 0.9 | 13.4 | 2.8 |
| Golabi-Pishva | Tahran | 129 | 7 | 3 | 3 | 5 | 1 | 5 | 2 | 3 | 3 | 3 | 5 | 1 | 9.3 | 18.5 | 1016.0 | 0.6 | 15.4 | 3.3 |
| Siah-Daneh-Kan | Tahran | 130 | 7 | 3 | 3 | 3 | 1 | 5 | 2 | 2 | 1 | 3 | 3 | 5 | 49.9 | 44.2 | 1420.8 | 0.8 | 15.5 | 3.1 |
| Toughi-Varamin | Tahran | 131 | 7 | 3 | 3 | 3 | 1 | 5 | 2 | 1 | 1 | 3 | 3 | 5 | 20.1 | 33.2 | 1161.3 | 0.7 | 13.4 | 3.1 |
| Narak-Kan | Tahran | 132 | 3 | 3 | 3 | 3 | 1 | 5 | 2 | 2 | 1 | 3 | 3 | 5 | 46.8 | 46.9 | 1355.0 | 0.7 | 17.0 | 2.4 |
| Sabz-Varamin | Tahran | 133 | 7 | 3 | 3 | 3 | 2 | 5 | 2 | 1 | 1 | 3 | 3 | 2 | 3.8 | 45.2 | 2011.2 | 0.7 | 15.0 | 2.4 |
| Shirin-Kan | Tahran | 134 | 7 | 3 | 3 | 3 | 1 | 5 | 2 | 3 | 2 | 3 | 5 | 3 | 13.9 | 26.9 | 1345.2 | 0.5 | 15.9 | 3.7 |
| Vahshi-Kan | Tahran | 135 | 7 | 3 | 3 | 3 | 1 | 5 | 2 | 1 | 2 | 3 | 3 | 4 | 34.5 | 46.9 | 1416.5 | 0.9 | 15.6 | 2.1 |
| Ghahveh-Daneh-Kan | Tahran | 136 | 7 | 3 | 3 | 3 | 3 | 5 | 2 | 2 | 1 | 3 | 3 | 2 | 5.7 | 50.5 | 1859.0 | 1.3 | 13.0 | 3.2 |
| Shahpar-Pishva | Tahran | 137 | 7 | 5 | 3 | 3 | 1 | 5 | 2 | 1 | 1 | 3 | 5 | 5 | 2.4 | 50.7 | 1073.0 | 1.0 | 8.8 | 3.2 |
| Summary name | Origin | code | Number of fruits in tree | fruit mean weight | tree mean yield | Anthocyanin on Branch of This year | Petiole Color | Fruitful Flower Size | Fruit Albedo Color | Fruit Bottom shape | Fruit Heel shape | Juicy of Arils | Seed Size | Seed Color | Total Anthocyanins (mg/g) | Antioxidant Activity (%) | Total Polyphenols (mg/l) | Acidity (g/l) | Total Soluble Solid(%) | pH |
| Shahpar-Varamin | Tahran | 138 | 7 | 3 | 3 | 5 | 1 | 5 | 2 | 3 | 2 | 3 | 3 | 3 | 14.0 | 27.8 | 961.5 | 1.5 | 13.4 | 3.5 |
| Bi-Nam | Unknown | 139 | 7 | 5 | 3 | 3 | 1 | 5 | 2 | 1 | 1 | 3 | 3 | 5 | 43.9 | 49.6 | 1812.0 | 1.5 | 16.7 | 3.2 |
| Bi-Nam | Unknown | 140 | 7 | 3 | 3 | 3 | 2 | 5 | 2 | 3 | 3 | 3 | 5 | 1 | 2.6 | 48.8 | 1715.7 | 0.9 | 15.8 | 3.4 |
| Ghermez-Harabarjan | Yazd | 141 | 7 | 3 | 3 | 3 | 1 | 5 | 2 | 2 | 1 | 3 | 3 | 2 | 2.1 | 47.3 | 1907.7 | 0.6 | 13.2 | 2.6 |
| Garch-Shahvar | Yazd | 142 | 7 | 5 | 3 | 3 | 3 | 5 | 2 | 1 | 1 | 3 | 5 | 5 | 30.9 | 29.4 | 2357.5 | 0.5 | 14.9 | 2.2 |
| Tough-Gardan | Yazd | 143 | 7 | 5 | 3 | 3 | 3 | 5 | 2 | 1 | 1 | 3 | 5 | 2 | 2.3 | 18.9 | 975.3 | 0.9 | 14.5 | 3.2 |
| Zagh | Yazd | 144 | 7 | 5 | 3 | 3 | 1 | 5 | 2 | 2 | 1 | 3 | 5 | 5 | 2.4 | 16.2 | 858.0 | 0.9 | 15.6 | 3.8 |
| Shirin-Shahvar | Yazd | 145 | 7 | 5 | 3 | 3 | 2 | 5 | 2 | 3 | 2 | 3 | 5 | 3 | 5.8 | 23.4 | 1083.0 | 0.6 | 15.0 | 3.5 |
| Malas-Yazdi | Yazd | 146 | 7 | 3 | 3 | 3 | 1 | 5 | 2 | 1 | 1 | 3 | 5 | 5 | 43.4 | 37.9 | 1303.5 | 0.8 | 16.1 | 3.1 |
| Shirin-Harati | Yazd | 147 | 7 | 5 | 3 | 3 | 1 | 5 | 2 | 3 | 3 | 3 | 3 | 1 | 40.5 | 41.7 | 1314.3 | 1.1 | 13.9 | 2.9 |
| Aban-Mahi | Yazd | 148 | 7 | 3 | 3 | 3 | 1 | 5 | 2 | 2 | 1 | 3 | 5 | 5 | 46.9 | 24.6 | 1333.3 | 0.6 | 15.5 | 3.1 |
| Zagh-Harabarjan | Yazd | 149 | 7 | 3 | 3 | 3 | 1 | 5 | 2 | 1 | 1 | 3 | 5 | 5 | 21.8 | 29.0 | 1069.0 | 0.9 | 16.3 | 2.9 |
| Abarnadabad | Yazd | 150 | 7 | 3 | 3 | 3 | 2 | 5 | 2 | 2 | 1 | 3 | 5 | 2 | 14.1 | 11.1 | 269.5 | 0.5 | 14.3 | 3.0 |
| Summary name | Origin | code | Number of fruits in tree | fruit mean weight | tree mean yield | Anthocyanin on Branch of This year | Petiole Color | Fruitful Flower Size | Fruit Albedo Color | Fruit Bottom shape | Fruit Heel shape | Juicy of Arils | Seed Size | Seed Color | Total Anthocyanins (mg/g) | Antioxidant Activity (%) | Total Polyphenols (mg/l) | Acidity (g/l) | Total Soluble Solid(%) | pH |
| Marvast | Yazd | 151 | 7 | 3 | 3 | 3 | 1 | 5 | 2 | 3 | 2 | 3 | 5 | 3 | 0.2 | 36.7 | 1745.0 | 0.9 | 15.8 | 2.9 |
| Ghermez-Harati | Yazd | 152 | 7 | 3 | 3 | 3 | 1 | 5 | 3 | 1 | 1 | 3 | 3 | 5 | 23.6 | 72.4 | 1985.0 | 1.0 | 16.3 | 2.9 |
