# Supplementary material for: First assessment of Iranian pomegranate germplasm using targeted metabolites and morphological traits to develop the core collection and modeling of the current and future spatial distribution under climate change conditions
Source: PLoS One. 2023 Feb 3;18(2):e0265977. doi: 10.1371/journal.pone.0265977 (PMC9897574; doi:10.1371/journal.pone.0265977)
Supplement: Attachment — Submitted filename: SABRAO-J-Breed-Genet-533-352-366-MOTYLEVA.pdf [file pone.0265977.s005.pdf]

RESEARCH ARTICLE

SABRAO Journal
of Breeding and Genetics
53 (3) 352-366, 2021

LEAF MICROMORPHOLOGICAL AND BIOCHEMICAL FEATURES OF SCAB DISEASE IN IMMUNE AND MODERATELY RESISTANT COLUMNAR APPLE (*Malus domestica*) CULTIVARS

S.M. MOTYLEVA*, S.M. MEDVEDEV, N.G. MOROZOVA and I.M. KULIKOV

Federal Horticultural Research Center for Breeding, Agrotechnology and Nursery, Moscow, Russia
*Corresponding author email: motyleva_svetlana@mail.ru
Email addresses of co-authors: mos_vstisp@mail.ru, burmenko_j@mail.ru, vstisp@vstisp.org

SUMMARY

This research aimed to study the leaf micromorphological and biochemical features of columnar apple cultivars that are immune and moderately resistant to *Venturia inaequalis*, the causal agent of scab disease, during 2017–19 at the Federal Horticultural Research Center for Breeding, Agrotechnology, and Nursery, Moscow, Russia. Results revealed that the immune cultivars showed a higher level of adaptability to the fungus *V. inaequalis* than the moderately resistant cultivars. The large number of leaf trichomes on the abaxial leaf surface that confer protection was characteristic of the immune cultivars. The immune columnar apple cultivars had 6% fewer leaf stomata than the moderately resistant ones. The stomata of the immune cultivars were placed high in the leaf surface, were surrounded by chords and folds, and had high cuticular peristomatal rings that provided additional protection by preventing pathogen entry. Inclusions with parallelogram and rounded-star shapes were found in the cross-section cut of the central leaf midrib of the immune cultivars. By contrast, the inclusions were mostly rounded-star-shaped in moderately resistant cultivars. Analysis through electronic microscopy with energy dispersion spectrometry revealed that the Ca and K contents in the parallelogram-shaped inclusions were 3 and 2 times higher than those in the rounded-star-shaped inclusions, respectively. In terms of ash composition, the P, Mg, and Ca contents in the leaves of immune columnar apple cultivars were 1.5, 1.5, and 1.6 times higher than those in the leaves of moderately resistant cultivars. The ultrafine surface study was more reasonable than other methods for identifying the phytopathogen resistance of fruit trees. The use of modern instrumental analytical methods for the complex evaluation of fruit crop leaves to reveal adaptations to biotic factors is meaningful for breeding use.

Keywords: *Malus domestica* Borkh., *Venturia inaequalis*, leaves, micromorphology, mineral composition, metabolites

Key findings: The micromorphological features of the surface; the number of stomata; the nature and chemical composition of inclusions, ash elements, and carbohydrates varied significantly in the leaves of columnar apple cultivars with immunity and moderate resistance to scab.

Manuscript received: June 5, 2021; Decision on manuscript: July 20, 2021; Accepted: August 1, 2021.
© Society for the Advancement of Breeding Research in Asia and Oceania (SABRAO) 2021

Communicating Editor: Dr. Gwen Iris Descalsota-Empleo

INTRODUCTION

Domesticated apple (*Malus domestica* Borkh.) is the most important fruit crop in the temperate zone and has a total annual yield of 89.3 million tons (FAOSTAT, 2020). Apple scab is one of the most harmful diseases of this fruit tree, and its spread in apple-growing regions has led to significant economic losses in production (70%–100%) (Biggs, 1990; Becker, 1993; Yakuba, 2013). Apple scab can be controlled by following the agrotechnique rules of cultivation in combination with the use of fungicides and resistant cultivars (Doroshenko, 2012).

The fungus *Venturia inaequalis* (Cooke) G. Winter is the pathogenic agent of apple scab. The information on the scab resistance of apple varieties and hybrids with the Rvi6 (Vf) and Rvi5 (Vm) genes has been provided by past research (Gessler and Stumm, 1984; Valsangiacomo and Gessler, 1988; Chevalier *et al*., 1991; Le-Cam *et al*., 2002; Lacis, Ikase and Rungis, 2011; Kozlovskaya *et al*., 2018). Russian studies focused on the epidemiology of scab (Fedorova, 1977; Smolyakova and Yakuba 2003; Yakuba, 2013), the genetic mutation and variability of *V. inaequalis* (Barsukova, 1991; Zhdanov and Zhuk, 2006), the genetics of plant–pathogen interaction, and breeding for disease resistance (Zhdanov and Sedov, 1991; Kichina, 2006; Sedov *et al*., 2009; Savelyeva and Savelyev, 2012). However, only few studies have been devoted to the physiological and biochemical aspects of the pathogenicity of *V. inaequalis*.

Studies on plant cuticle functions demonstrated the importance of the plant cuticle in resistance to colonization by microorganisms (Martin and Juniper, 1970). However, the authors did not clearly specify whether the cuticle plays a limiting role in plant protection concerning parasite–host interactions or whether cuticle functions could be the main factors in resistance to pathogen penetration into the leaves (Pcries, 1962; Roberts and Martin, 1963). Certain chemical substances and elements may participate in the mechanisms of plant protection systems. Secondary metabolites build a natural protection mechanism (Petkovsek *et al*., 2011; Kviklys *et al*., 2014). Plant resistance to biotic and abiotic stressors depends on the synthesis of physiologically active antioxidant substances that prevent oxidative stress in photosynthetic organs (Bonarska-Kujawa *et al*., 2011; Ceymann *et al*., 2012; Hua *et al*., 2014). Metabolic sugars influence resistance to biotic stresses (Morkunas and Ratajczak, 2014). A previous analysis confirmed that rapidly changing environmental factors are constant motivators of inner reactions in plants. The integrated approach and use of instrumental research methods allow sorting out low-value plants at the first stages of breeding, thus reducing workload and expediting the breeding process. Furthermore, breeding for disease resistance is a promising way to cope with the scab disease caused by the fungus *V. inaequalis*.

The compact forms of columnar apple trees that quickly start fruiting and exhibit a healthy exterior with good leaf coverage have an essential practical interest (Savelyev, 1998). As a result of the completion of breeding programs for apple columnar varieties created on the basis of the compatibility gene, new columnar apple cultivars have been created and recommended for cultivation in many countries, including Russia. However, such genotypes are not widely planted in industrial plantations but are instead used in individual and farm horticulture (Kichina, 2006; Savelyeva and Savelyev, 2012).

This study aimed to analyze the lowest number of morphological and biochemical features, i.e., leaf surface shape, stoma form and size, and cuticle thickness in combination with the ash composition and metabolic analyses to

differentiate the leaves of scab-immune and moderately resistant columnar apple cultivars.

MATERIALS AND METHODS

Experimental conditions

The leaves of eight columnar apple cultivars were studied during 2017–19 at the Federal Horticultural Research Center for Breeding, Agrotechnology, and Nursery, Moscow, Russia. The scab-immune columnar apple cultivars were 'Valuta', 'Lukomor', 'Senator', and 'Triumph', whereas the moderately resistant cultivars were 'Vasyugan', 'Ostankino', 'Chervonets', and 'President' (Table 1). Fully formed leaves were studied (Figure 1). The selection of leaves was performed in mid-June. The columnar apple trees were grown in the laboratory plot. The study area has a temperate continental climate. Its height above sea level is 168 m, and its coordinates are 55° 56′ north latitude, 37° 64′ east longitude. The morphological and biochemical features of fully formed leaves were studied during mid-June.

Data recorded

The determined parameters included morphological and biochemical features, i.e., the microstructure of the adaxial and abaxial leaf surfaces, the number and size of stomata, the thickness of the cuticle layer, and the mineral and metabolite composition of the leaves. These features were determined via analytical raster electron microscopy with a REM JEOL JSM–6010 LA (JEOL Ltd, Japan). The quality composition of metabolites present in the leaves of columnar apple trees was determined through gas chromatography–mass-spectrometry (GCMS) by using the instrument JEOL JMS-Q1050GC (JEOL Ltd, Japan).

Chemicals

All the reagents, solvents, and standards used were of analytical quality (minimal purity 99%) and were bought from Sigma Aldrich, USA.

Table 1. Origins and originators of the apple cultivars under study.

Apple cultivars	Origin	Authors
Apple cultivars with scab immunity based on *Rvi6* (*Vf*) gene		
Valuta	Columnar apple elite KV6 × with the scab-immune donor OR38T17	Kichina V.V., Morozova N.G.
Lukomor	From breeding column-like apple elite KV6 × the scab-immune donor OR38T17	Kichina V.V., Morozova N.G.
Senator	From hybridization of donors VM41497 × KV103 (columnarness)	Kichina V.V, Morozova N.G..
Triumph	From the column-likeness donor KV5 seeds pollinated by the scab-immune donor D103-189 (F4 from M. Floribunda). The pollen was sent by Prof. E.V. Williams from Purdue University, Indianna, USA (310 /3)	Kichina V.V., Morozova N.G.
Apple cultivars with moderate resistance to scab		
Vasyugan	From cross breeding of the columnar donor KV5 × Brusnichnoe (376/106)	Kichina V.V., Morozova N.G.
Ostankino	From cross breeding of Obilnoe × Vazhak (KB71)	Kichina V.V.
Chervonets (with*Rvi5* (*Vm*) gene)	From cross breeding of the American donor SR0523 × Vazhak (KB82)	Kichina V.V., Morozova N.G.
President	Seedlings from free pollination × column-like donor KV103	Kichina V.V., Morozova N.G.

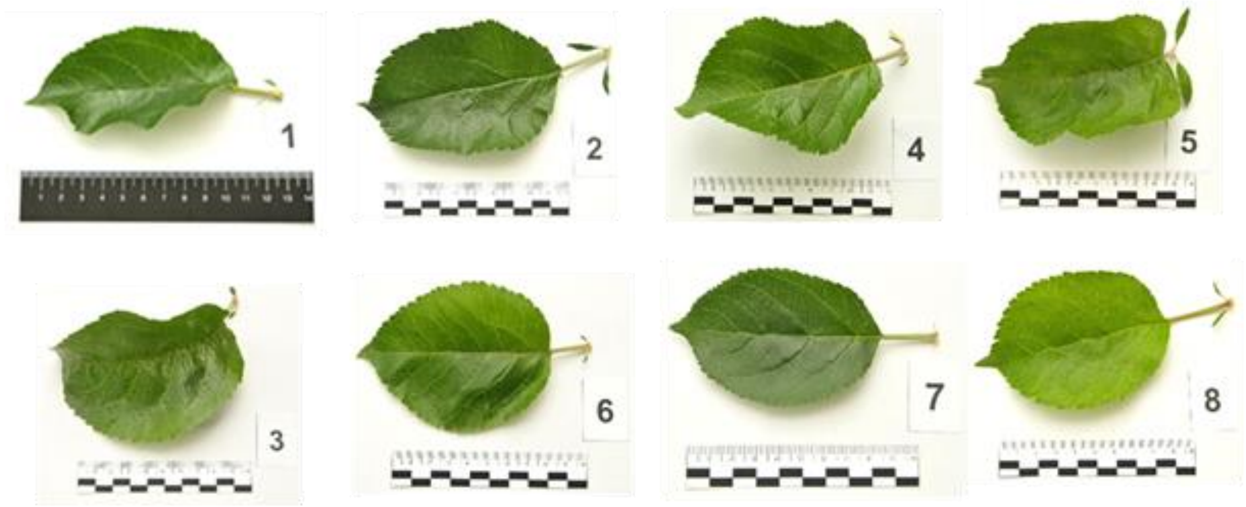

Figure 1. Apple cultivar leaves.
Immune apple cultivars – 1: Valyuta, 2: Lukomor, 4: Senator, 5: Triumf.
Moderately resistant apple cultivars – 3: Chervonets, 6: Ostankino, 7: President, 8: Vasyugan.

Visualization of the leaf surface and cross-section cuts

For examining the morphology of the abaxial and adaxial leaf surface, sections with dimensions of 5 mm × 5 mm were taken from the left and right of the central midribs of 10 leaves and placed on carbonic scotch mounted on the microscope object table. The leaf cross-sections were 0.5–1 mm thick and were fixed on the stitching base. Leaves were not subjected to preliminary processing because microscopy was conducted under low vacuum (60 Pa), and the deformation of the cross-section cuts was not significant.

Leaf processing for energy dispersion spectrometry

Fresh vegetal material, i.e., leaves with an average mass of 10 g, were dried in a drying cupboard at 80 °C until air-dried. The dried samples were mineralized in a muffle furnace (Naberterm, Germany) at T = 400 °C. The obtained d ash was dispersed by ultrasound at 18 kHz frequency for 15 min. An even layer of the dispersant was applied on the object table covered with carbonic scotch.

Leaf processing for GCMS analysis

The middle layers of the leaves were used for this analysis. Fresh leaves were weighed with a homogenizer IKA (Germany). A total of 0.5 g of the leaves was extracted with 15 ml of pure ethanol and centrifuged with Sigma 3-18 KHS (Germany). A total of 200 ml of the centrifugate was evaporated to dryness under helium flow. The derivation was performed with N,O-Bis (trimethylsilyl) trifluoroacetamide (BSTFA) for trimethylsilylation in accordance with the method described by Lebedev (2003). BSTFA was used for silylation within 30 min at 100 °C.

Energy dispersion spectrometry of mineral elements

The mineral (ash) composition was determined via energy dispersion spectrometry with an energy dispersion spectrometry (EDS) analyzer combined with the scanning electron microscope

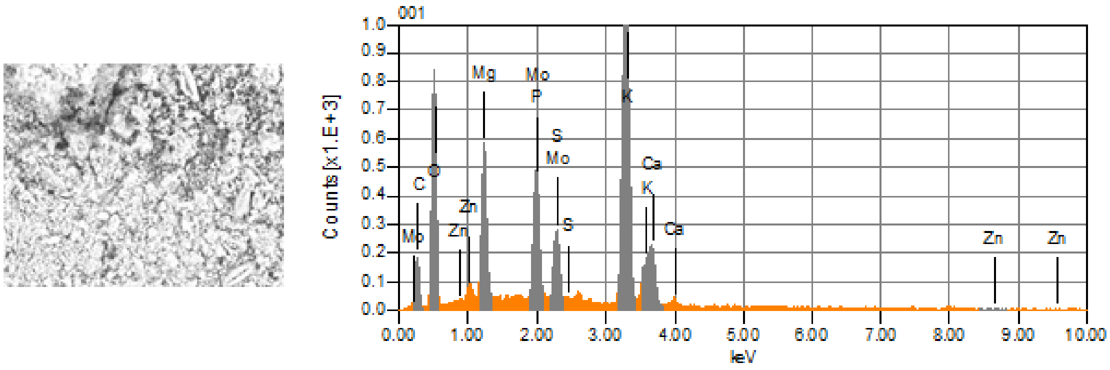

Chemical formula	mass%	Atom%	Sigma	K ratio	Line
C	64.93	71.39	0.02		K
O	25.69	21.20	0.05		K
Mg	6.39	5.21	0.11		K
P	5.73	4.31	0.11		K
S	2.20	1.08	0.17		K
K	26.77	14.26	0.01		K
Ca	5.49	2.16	0.11		K
Zn	0.13	0.04	0.21		K

Figure 2. EDS analysis: studied area and spectrogram.

JEOL JSM 6090 LA (Japan) in accordance with the methodology of Motyleva (2018) and Motyleva *et al*. (2021). Spectra and element distribution data were obtained together with images on a raster electron microscope. Elemental composition was evaluated on the basis of the weight percentage of 12 elements, namely, Mg, Si, P, S, K, Ca, Cr, Mn, Fe, Cu, Zn, and Se, which were reliably diagnosed. EDS was used for the qualitative and quantitative analysis of the existing elements in the X-ray spectra acquired through the electronic beam scanning of the observed image. X-ray microanalysis data were obtained in accordance with standard protocols and included the microstructure image of the sample under study, the table of the data on weight and atomic correlations, spectra, and histograms. An example of the spectral data is shown in Figure 2. Ten measurements were taken for each ash sample. The local analytical area was 3 mm, and the scanning area was at least 12 µm. The average quadratic deviation did not exceed 1.2%–6.9%. The analytical observations were performed in triplicate.

Metabolic analysis via GCMS

Metabolites analysis was performed with a JMS-Q1050GC chromatograph via GCMS. A DB-5HT capillary column (Agilent, USA) with a length of 30 m, inner diameter of 0.25 mm, film thickness of 0.52 um, and helium gas carrier was used. Substances were identified through comparison with the values and mass spectra of NIST-5 provided by the National Institute of Standards and Technology Library, USA. The scanning range was 33 m/z to 900 m/z. The probability of substance determination was within 75% to 98%. The temperature gradient during the analysis was 40 °C to 280 °C. The oven temperature was ramped up from 40 °C to 130 °C at a rate of 1 °C min^{-1}, from 130 °C to 200 °C at 2 °C min^{-1}, and from 200 °C to 280 °C at 4 °C min^{-1} and held at 280 °C for 40 min. The temperature of the ion source was −200 °C. Gas flow (helium) in the column was equal to 2.0 mL min^{-1}. Split-flow injection mode was used. The sample injection volume was 1–2 mcl of the evaporated extract.

Statistical analyses

All the analyses were performed in triplicate, and the results were expressed as mean values (n = 3) with standard deviation. The Excel package (Microsoft Excel, v. 2016) was used for statistical analysis.

RESULTS

Scanning electron microscopy

The majority of the columnar apple cultivars had smooth adaxial leaf surfaces that were fully covered with a thick tuberculate or folded wax layer and lacked hairs (trichomes) and stomata. The microstructure of the leaf surface is a varietal character and depends on the cultivar. Trichomes were rarely found on the adaxial leaf surface of the immune apple cultivar 'Lukomor' (Figure 3).

The whole abaxial leaf surface of the immune columnar apple cultivars, as observed in the apple cultivar 'Valyuta', was thickly covered with long, flexuose, interlacing round, sometimes flattened trichomes (Figure 4-1). The number of trichomes on the abaxial leaf surface in the moderately resistant apple cultivars, as exhibited by the apple cultivar 'President' (Figure 4-6), was visibly lower than that in the immune cultivars. In the immune columnar apple cultivars, the stomata were large and elevated, and the stomatal rings were high, which protected the stomatal pore (Figures. 4-1a, 4-2, 4-3). The stomata of the moderately resistant apple cultivars were less elevated than those of immune cultivars (Figure 4-6a). The immune columnar apple cultivars presented a lower number of stomata (average of 385.5 pcs/mm^2) than moderately resistant apple cultivars (408.3 pcs/mm^2) (Figure 5). Scab progression along with fungal implantation and areas of destruction in the cuticle were found on the leaves of the apple cultivar 'President'.

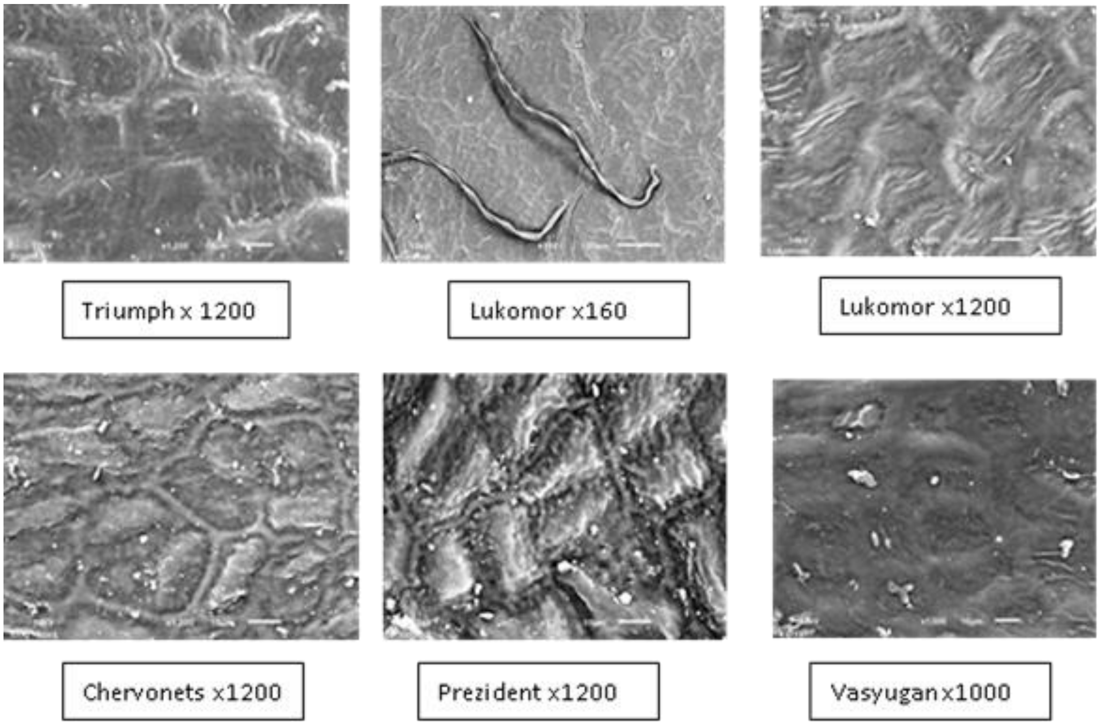

Figure 3. Microstructures of the adaxial surfaces of apple leaves under 160×, 1000×, and 1200× magnification.

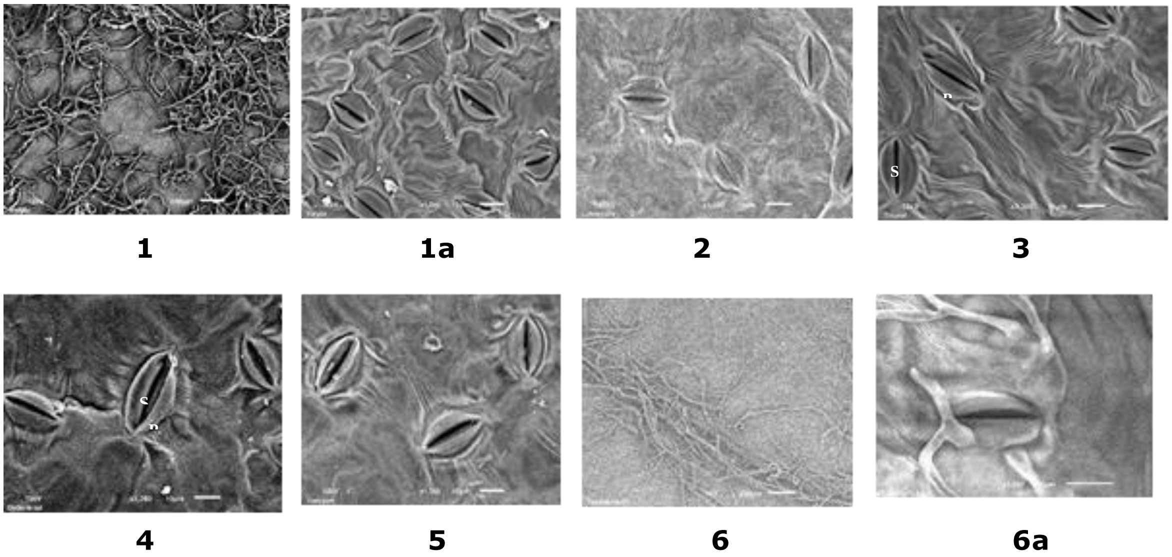

Figure 4. Trichomes (T), stomata (S), peristomatal rings (P), and abaxial leaf sides of apple cultivars. 1, 1a –Valyuta, 2 – Lukomor, 3 – Triumf, 4 – Ostankino, 5 – Vasyugan, 6 and 6a – President.

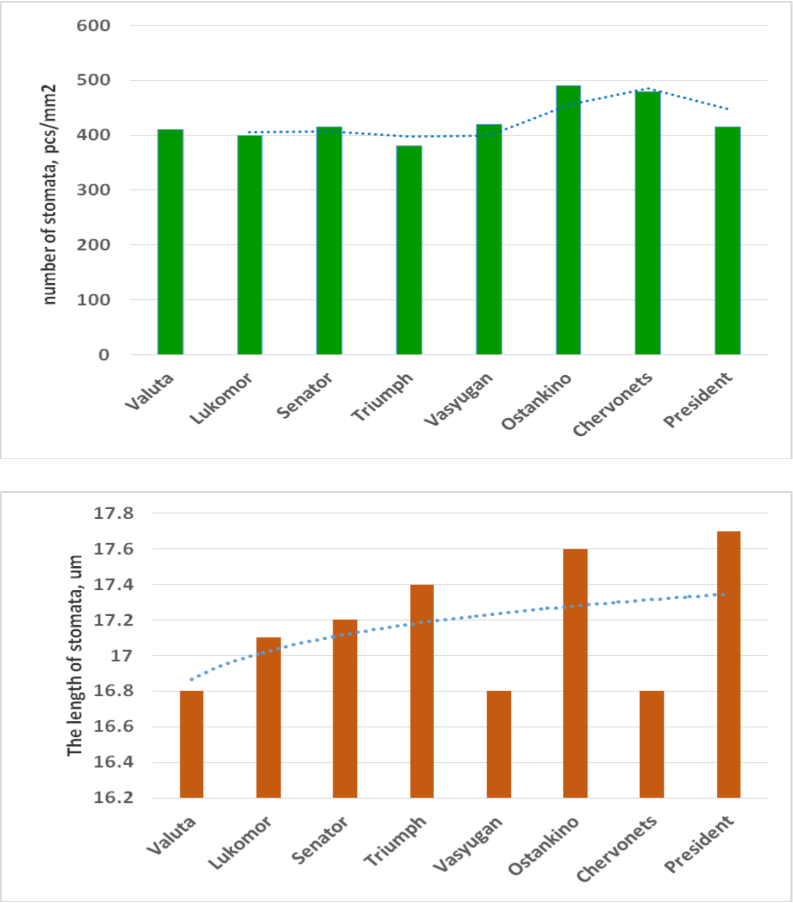

Figure 5. Comparative morphometric indicators of immune and moderately resistant apple cultivars to scab, up – several stomata (pcs/mm^2), down – the length of stomata (μm).

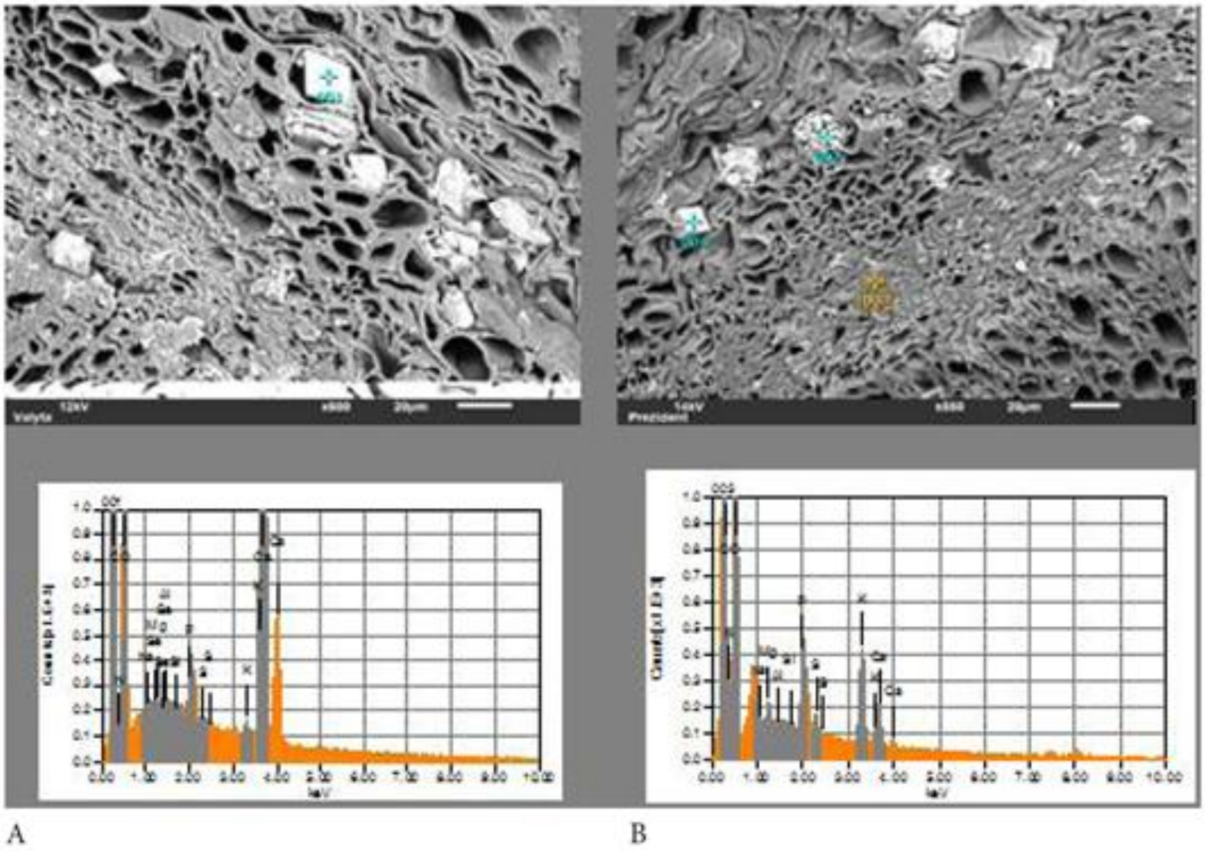

Figure 6. Crystals in the cross-section cuts of the leaf central midrib. A – Valyuta, immune cultivar; B – President, moderately resistant cultivar. The points for microanalysis processing are marked with parallelograms or rounded-star shapes.

In the cross-section cuts of the central midrib, numerous inclusions (metabolite products) had accumulated in the form of granules and crystals. Local EDS analysis showed that the inclusions contained Ca (5.9–8.3 mass%); K (0.34–0.38 mass%); and S, P, Mg, and Na with 0.03–0.07 mass% (Figure 6). The Ca and K contents of the parallelogram-shaped inclusions that were found in large numbers on the leaves of immune apple cultivars were respectively 3 and 2 times higher than those of the rounded-star-shaped inclusions that were found mostly on the leaves of moderately resistant apple cultivars.

Thus, the morphological and biochemical features of immune apple cultivars indicated a higher level of adaptability to the biotic factors of the environment (scab) than moderately resistant cultivars and were correlated with the reference data. The immune apple cultivars had the following characteristics: 1) a highly developed wax layer, 2) a large number of trichomes (hairs) on the abaxial leaf surface with protective functions, 3) a lower number of stomata (on average on 6%) than moderately resistant apple cultivars, 4) chords and folds around the stomata, 5) well-developed peristomatal rings providing additional protection, and 6) two types of inclusions: parallelogram-shaped and rounded-star-shaped. In the moderately resistant apple cultivars, the inclusions were mostly rounded-star-shaped.

Ash composition

Two leading factors, genetic and environmental (the processes of absorption and deposition of chemical elements), influence elemental composition in plants. The cumulative amount of ash elements in the leaves of immune cultivars was 1.3 times higher than that in the leaves of moderately resistant cultivars (Table 2). Analyzing the average values for each element under study revealed that the leaves of the immune apple cultivars contained 1.5 times more P and Mg, 1.2 times more K, and 1.6 times more Ca than the leaves of moderately resistant cultivars. K accounted for the major proportion of the leaf ash composition and could reach up to 40.5% (Table 3). Scientific literature showed that K is very mobile in the plants and acts as an osmotic agent in stomatal opening and closing. Mn is a part of the chlorophyll molecule and is involved in several enzyme systems. P is found in phospholipids, nucleoproteins, and macroenergy connections between phosphate groups and serves as the main energy transfer agent in plants. S is a component of cystine, cysteine, other amino acids, biotin, thiamine, and coenzymes. It is less mobile than P and K. Ca is found in cell walls in the form of calcium pectate, which affects cell wall elasticity. The important role of these ash elements has also been noted in plant adaptive processes (Cakmak, 2005; Maathuis, 2009). Certain oligo-elements (Mg, Cr, Fe, Cu, Zn, and Se) were present with the range of 0.01 (Cr and Fe) to 0.25 (Cu) mass%.

Metabolic analysis

The composition analysis of the main substances contained in the ethanol extract prepared from columnar apple cultivar leaves was determined via GCMS. In total, 40 metabolites were identified. Their peak height was not less than 0.1% of the instrument scale. The metabolites were categorized into the following groups: compounds possessing antimicrobial properties (seven compounds); other organic compounds (nine compounds); and sugars and their derivatives (24 compounds).

Among the seven compounds with antimicrobial properties, five were organic acids possessing antimicrobial properties (3-hydroxybutyric, malic, α-linolenic, gluconic, and acrylic acids) and the other two were the triatomic alcohol glycerol and the highly biologically active triterpenoid lupeol (Table 3). The amount of glycerol in the leaves of the immune apple cultivars was the highest and was 5 times more than that in the leaves of moderately resistant cultivars. The malic acid and acrylic acid contents of the leaves of immune cultivars were 4 and 1.6 times higher, respectively, that those of the leaves of moderately resistant cultivars. Lupeol was detected only in the leaves of immune apple cultivars.

Among the nine other organic compounds, five were organic acids (2-butanedioic, monoethyl malonic, succinic, fumaric acid, and 2-ketoglutaric acid) and three were saturated fatty acids (Table 4). The content of succinic acid was 5 times higher in the leaves of columnar immune apple cultivars than in the leaves of moderately resistant cultivars. Fumaric, 2-propenoic, 1, 2-ketoglutaric, and stearic acids were detected only in the leaves of immune cultivars.

Among the carbohydrates, fructose had the highest proportion. Its content in the leaves of immune cultivars was twice that in the leaves of moderately resistant cultivars. Notably, 21 out of 24 carbohydrates were observed in the leaves of immune apple cultivars, only 11 compounds were detected in the leaves of moderately resistant cultivars, and eight compounds accounted for the total carbohydrates (Table 5).

DISCUSSION

In this study, micromorphological and biochemical analyses on scab-immune and moderately resistant apple cultivar leaves were performed. The micromorphological

Table 2. Ash content of apple cultivar leaves, mass% in ashes, and $\bar{x}$ (2017–2019).

Elements	Immune to scab				Moderately resistant to scab			
	Valyuta	Chervonets	Triumph	Average	President	Ostankino	Senator	Average
Mg	5.73	5.39	5.04	5.39	3.34	3.04	3.53	3.31
Si	0.65	0.68	0.62	0.65	0.44	0.51	0.61	0.52
P	5.46	5.28	5.91	5.55	4.32	1.18	3.64	3.05
S	0.44	0.47	0.49	0.47	0.36	0.34	0.38	0.36
K	41.27	40.59	39.42	40.43	31.36	33.56	32.81	32.58
Ca	0.45	05 1	0.45	0.45	0.29	0.2 3	0.28	0.29
Cr	0.03	0.01	0.02	0.02	0.01	0.01	0.04	0.02
Mn	0.04	0.04	0.04	0.04	0.02	0.01	0.05	0.03
Fe	0.04	0.04	0.01	0.03	0.01	0.02	0.02	0.02
Cu	0.02	0.01	0.25	0.11	0.09	0.01	0.11	0.07
Zn	0.04	0.06	0.05	0.07	0.04	0.05	0.04	0.06
Se	0.05	0.085	0.105	0.08	0.04	0.066	0.05	0.052
∑	52.84	54.37	52.73	53.46	40.48	38.91	41.7	40.46

Table 3. Antimicrobial compounds detected in the leaf extracts of apple cultivars with immunity or moderate resistance to *V. inaequalis.*

No.	T_{min}	Metabolite, Molecular Formula	Peak height, % of scale		Biological characteristic
			Immune	Moderately resistant	
1	13:49	Glycerol, $C_3H_8O_3$	80	7–15	Antimicrobial
2	10:25	3-Hydroxybutyric acid, $C_4H_8O_3$	2	2	Antimicrobial
3	16.34	Malic acid, $C_4H_6O_5$	40–60	8–10	Antimicrobial
4	24:09	α-Linolenic acid, $C_{18}H_{30}O_2$	0.2–0.3	0.05–0.1	Polyunsaturated fatty acid, antimicrobial
5	19:38	Gluconic acid, $C_6H_{12}O_7$	10–15	8–20	Antimicrobial
6	21.52	Acrylic acid, $C_3H_4O_2$	4.0–5.2	0.2–2.5	Antimicrobial
7	39:26	Lupeol, $C_{30}H_{50}O$	1–1.2	–	Antimicrobial

Table 4. Organic compounds detected in the leaf extracts of apple cultivars with immunity or moderate resistance to *V. inaequalis.*

No.	T_{min}	Metabolite	Peak height, % of scale		Biological classification
			Immune	Moderately resistant	
1	10:49	2-Butanedioic acid, $C_4H_6O_4$	0.2–1.5	0.1–1.0	Organic acid
2	11:29	Monoethyl malonic acid, $C_5H_8O_4$	8–10	5–10	Organic acid
3	14.04	Succinic acid, $C_4H_6O_4$	11–15	3–4	Organic acid
4	16.46	Fumaric acid, $C_4H_4O_4$	0.1–0.5	–	Organic acid
5	25:00	2-Propenoic acid, $C_3H_4O_2$	0.1–0.3	–	Organic acid
6	17:54	1,2-Ketoglutaric acid, $C_6H_{12}O_7$	0.2–0.4	–	Keto acid
7	18.24	Lauric acid, $C_{12}H_{24}O_2$	0.2–0.4	0.1	Saturated fatty acid
8	22:30	Palmitic acid, $C_6H_{32}O_2$	0.1	0.05–0.1	Saturated fatty acid
9	24:19	Stearic acid $C_{18}H_{36}O_2$	1–1.4	–	Saturated fatty acid

Table 5. Sugar derivatives detected in the leaf extracts of apple cultivars with immunity or moderate resistance to *V. inaequalis.*

No.	T_{min}	Metabolite	Peak height, % of scale		Biological classification
			Immune	Moderately resistant	
1	20:18	Arabinofuranosem, $C_5H_{10}O_5$	1–1.1	–	Monosaccharide
2	16.21	β-DL-arabinopyranosem, $C_5H_{10}O_5$	0.8–1.2	–	-do-
3		D-Erythro-pentofuranosem, $C_5H_{10}O_5$	1.0–1.2	–	-do-
4	17.06	D-(−)-Ribofuranose,, $C_5H_{10}O_5$	–	0.2	-do-
5	18.22	Glucofuranoside, $C_6H_{12}O_6$	1.0–1.2	0.2–0.4	-do-
6	19.47	Methyl-D-glucoside,, $C_7H_{14}O_6$	0.8–1.1	0.1–0.4	-do-
7	19.58	D-(+)-Xylose, $C_5H_{10}O_5$	4–5	0.05–0.1	-do-
8	25.53	Levoglucosan, $C_6H_{20}O_5$	11–13	0.1–0.4	-do-
9	17.01	D-(−)-Ribofuranose, $C_5H_{10}O_5$	4–8	–	-do-
10	18.30 20:45	D-(−)-Tagatofuranose, $C_6H_{12}O_6$	2–3.5	0.4–0.5	-do-
11	19.01	Fructose, $C_6H_{12}O_6$	45–50	15 –35	-do-
12	20.38	D-Psicofuranose, $C_6H_{12}O_6$	20–23	12–30	-do-
13	20:55	D-Allofuranose, $C_6H_{12}O_6$	7–8	–	-do-
14	20.55	D-(+)-Talofuranose, $C_6H_{12}O_6$	0.4–0.6	0.1–0.2	-do-
15	22.20	Glucopyranose, $C_6H_{12}O_6$	-	8–11	-do-
16	22:17	α-D-Mannopyranose, $C_6H_{12}O_6$	4–5	–	-do-
17	19.23	Arabinoic, acid, $C_5H_{10}O_6$	0.5–1.2	–	Sugar acid
18	22:07	Sorbitol, $C_6H_{14}O_6$	0.8–1.2	–	-do-
19	19:29	Erythritol, $C_4H_{10}O_4$	0.4–0.8	–	Sugar alcohol
20	22.06	D-Mannitol, $C_6H_{14}O_6$	–	0.2–0.4	-do-
21	21:22	Gulonic, acid, g-lactone, $C_6H_8O_6$	3–3.8	–	Sugar acid lactone
22	31:03	Lactulose, $C_6H_{22}O_{11}$	4–4.5	–	Disaccharide
23	32:43	D-(+)-Turanose, $C_{12}H_{22}O_{11}$	8– 11	–	-do-
24	314:35	Sucrose, $C_{12}H_{22}O_{11}$	15–20	–	-do-

features of the *Malus* leaf epidermis have been studied in past but not amply (Potter *et al*., 2007; Takhtajan, 2009; Volonkov *et al*., 2020). Information on the micromorphological features of the leaf epidermis can be very useful when choosing resistant genotypes for predicting the characteristics of developed hybrids (Kumachova *et al*., 2019). Riederer and Muller (2006) discovered that a leaf surface can be smooth or have folded surface microtopography, which can be seen only using scanning electron microscopy. The features of the leaf cuticular layer plays an essential role in the adaptation of plants to existing environmental conditions. It is connected to the regulation of guard cell aperture and morphological parameters, i.e., the number, size, and form of stomata on the leaf area (Miroslavov and Kravkina, 1990; Miroslavov, 1994; Royer, 2001, Hetherington and Woodward, 2003; Mizutani and Kanaoka, 2017).

The leaf surface micromorphology of different scab-immune apple cultivars has been studied before (Motyleva and Kuznetsov, 2010). The adaxial leaf surface of columnar apple cultivars was characterized by differently organized folded microtopologies and a well-developed wax layer. Moreover, pattern structure was found to be a cultivar-specific trait. On the abaxial leaf surface of immune apple cultivars, trichomes were located not only along the central midrib but also in the inter-rib space as well. In the stomatal zone, numerous chords that were the original folds of the cuticle were located surrounding the stomata; the stomata had high peristomatal rings. These morphological structures can stop pathogen entry. The trichomes of moderately resistant apple cultivars were

distributed sparsely, and the peristomatal rings were flattened. The present findings were highly correlated with past results showing that apple leaves have radially spreading peristomatal folds that essentially reduce epidermal wetting and provide increased resistance to pathogens (Ganeva and Uzunova, 2010; Pautov *et al*., 2014).

Waxes and cutin are endocrine substances, and secondary metabolites, terpenoids, volatile compounds, and other biologically active substances are synthesized in the cells (Evert and Eichhorn, 2006). The formation of Ca-containing crystals in different plant cells is also known (Ward and Olsvig-Whittaker, 1993; Volk *et al*., 2008). Modern studies have stated that Ca^{2+} is the key element of signaling pathways and is mobilized during interactions between plants and pathogens (Sanders *et al*., 2002; Reddy and Reddy, 2004; White, 2004). On the basis of the reference data, the morphological and biochemical results of this research revealed that physiological and metabolic processes in scab-immune and moderately resistant apple cultivar leaves occurred differently. The chords (original folds of the cuticle) and peristomatal rings formed with different intensities in the stomata zone. Ganeva and Uzunova (2010) and Pautov *et al*. (2014) revealed that apple leaves are characterized by two types of peristomatal folds that radiate in different directions, and girded guard cells that form peristomatal rings. Although the role of Ca-containing crystals is not fully understood, the leaves of immune apple cultivars had two types of crystals, i.e., parallelogram-shaped and rounded-star-shaped, whereas the moderately resistant cultivars, had mostly rounded-star-shaped inclusions. Such cuticular folds reduce epidermal wetting and provide increased resistance to pathogens. EDS analysis revealed differences in Ca content in these types of crystals. Ash content analysis showed that Ca content in the leaves of immune cultivars was higher than that in moderately resistant apple cultivars.

The scab resistance of apple leaves may be due to the synthesis of substances that decelerate the development of agents in exodermal cells (Gratzl *et al*., 2014; Nieto-Penalver *et al*., 2014; Ma *et al*., 2019). This research showed that the leaves of immune apple cultivars have larger quantities of the substances possessing antimicrobial activities than the leaves of moderately resistant ones. Past studies have demonstrated that sugars are very important in plant resistance to the diseases caused by microorganisms and have described the role of carbohydrates as signaling molecules in protective reactions (Rolland *et al*., 2006; Morkunas and Gimerek, 2007; Doehlemann *et al*., 2008; Rosa *et al*., 2009; Yu *et al*., 2010).

The results also revealed that the substances that participated in the quality analysis of carbohydrates were 1.9 times higher in the leaves of immune cultivars than in the leaves of moderately resistant ones. These results corresponded with past findings stating that pathogens cause changes in the quality composition of carbohydrates (Morkunas and Gmerek, 2007; Morkunas and Ratajczak, 2014). Disaccharides, i.e., lactulose, turanose, and sucrose, were detected only in the leaves of immune apple cultivars. The data of Wind *et al*. (2010) showed that sucrose acts as a signaling molecule. The morphological and biochemical findings revealed that physiological and metabolic processes occur differently in the leaves of cultivars with immunity or moderate resistance to apple scab.

CONCLUSIONS

Scanning electron microscopy revealed the folded surface of the cuticle, the presence of numerous trichomes, and the features of stomata configuration (the presence of chords and folds providing connection between the stomata and surrounding epidermis). Resistance to apple scab disease was speculated to depend on the microstructural features of the adaxial and abaxial leaf surfaces. The

results also showed that the genotype was the main factor that determined the content of primary and secondary metabolites. The leaves of immune apple cultivars with the *Rvi6* (*Vf*) gene exhibited a high Ca content. The Ca/K and Ca/Mg ratios of the leaves of immune apple cultivars were 3.1 and 5.2 times higher than those of the leaves of moderately resistant apple cultivars. The quality composition of secondary metabolites possessing antimicrobial activity and the number of mono- and disaccharides in the leaves of immune cultivars were higher than those in moderately resistant cultivars. Scanning electron microscopy, EDS, and GCMS were found to be useful for broadening our knowledge about the structural and biochemical features of apple leaves. The said approach has important significance in breeding processes because it enables the early diagnosis of scab resistance in apple genotypes.

REFERENCES

Barsukova ON (1991). Changes in the race composition of *Venturia inaequalis* (Cke.) Wint. in the Caucasus. *Mikologiya i fitopatologiya*. 25(6): 546-549 (in Russian).

Becker CM (1993). The cytology and histology of apple scab. In: A. R. Biggs, eds., Cytology, Histology and Histochemistry of Fruit Tree Diseases., ed. CRC Press, Boca Raton, FL, pp. 35-90.

Biggs AR (1990). Apple scab. In: Compendium of apple and pear diseases. A. L. Jones and H. S. Aldwinckle, eds., The American Phytopathological Society, St. Paul, MN., pp. 6-9.

Bonarska-Kujawa D, Cyboran S, Oszmianski J, Kleszczynska H (2011). Antioxidant properties of apple leaves and fruits extracts from apple leaves and fruits as effective antioxidants. *J. Med. Plants Res.* 5(11): 2339-2347.

Cakmak I (2005). The role of potassium in mitigating the harmful effects of abiotic stresses in plants. *J. Plant Nutr. Soil Sci*. 168(4): 521-530.

Ceymann M, Arrigoni E, Scharer H, Bozzi Nising A, Hurrell RF (2012). Identification of apples rich in health-promoting flavan-3-ols and phenolic acids by measuring the polyphenol profile. *J. Food Comp. Anal*. 26(1-2): 128-135.

Chevalier M, Lespinasse Y, Renaudin S (1991). A microscopic study of the different classes of symptoms coded by the Vf gene in apple for resistance to scab (*Venluria inaequalis*). *Plant Pathol.* 40: 249-256.

Doehlemann G, Wahl R, Horst RJ, Voll LM, Usadel B, Poree F, Stitt M, Pons-Kuhnemann J, Sonnewald U, Kahmann R, Kamper J (2008). Reprogramming a maize plant: transcriptional and metabolic changes induced by the fungal biotroph *Ustilago maydis*. *Plant J*. 56: 181-195.

Doroshenko TN (2012). The features of the fruit plants productivity potential realization in the years with weather anomalies. *Scien. J. KubSAU*. 82(8): 1-19.

Evert RF, Eichhorn SE (2006). Esaus plant anatomy: meristems, cells and tissues of the plant body: their structure, function and development, 3rd edn. John Wiley & Sons.

FAOSTAT (2020). Division, Food and Agriculture Organization of the United Nations. Statistics. Available online: http://www.fao.org/statistics/en/ (accessed on 1 February 2020).

Fedorova RN (1977). Apple scab. Leningrad: Kolos, pp. 64 (in Russian).

Ganeva T, Uzunova K (2010). Comparative leaf epidermis study in species of genus *Malus Mill*. (Rosaceae), *Bot. Serbica* 34: 45-49.

Gessler C, Stumm D (1984). Infection and stroma formation by *Venluria inaequalis* on apple leaves with different degrees of susceptibility to scab. *J. Phytopathol*. 110: 119-126.

Gratzl G, Paulik C, Hild, S, Guggenbichler JP, Lackner M (2014). Antimicrobial activity of polyacrylic acid block copolymers. *Mater. Sci. Eng*. 38: 94-100.

Hetherington AM, Woodward FI (2003). The role of stomata in sensing and driving environmental change. *Nature* 424: 901-908.

Hua Y, Guo H, Zhou X-G, Li X,Yang S, Song Y, Ma N, Chai C, Qiao X, Li L (2014). Correlations between soluble sugar and phenol contents in leaves and pear scab resistance. *J. Plant Pathol. Microbiol.* 5(1): 1-4.

Kichina VV (2006). Column-like Malus. Moscow. VSTISP. pp. 162.
Kumachova TK, Voronkov AS, Babocha AV, Rydchenko AS (2019). Morphofunctional characteristics of leaves and fruits in maloideae (Rosaceae): a microstructure of surface tissues. *Proc. Appl. Bot. Genet. Breed*. 180: 105-112.
Kviklys D, Liaudanskas M, Janulis V, Viškelis P, Rubinskienė M, Lanauskas J, Uselis N (2014). Rootstock genotype determines phenol content in apple fruits. *Plant, Soil, Environ*. 60(5): 234-240.
Kozlovskaya ZA, Kondratsenok Ju G, Gashenko TA, Yarmolich SA (2018). Identifi cation of new complex sources of resistance to apple diseases using molecular methods in Belarus. *J. Hort. Viticul*. 5: 23-29.
Lacis G, Ikase L and Rungis D (2011). Molecular characterization of the Latvian apple (Malus) genetic resource collection based on SSR markers and scab resistance gene Vf analysis. *Plant Genet. Resour: Characterization Util.* 9(2); 189–192.
Lebedev AT (2003). Mass spectrometry in organic chemistry. Tutorial. Publisher: BINOM. Lab knowledge; pp. 501. ISBN: 5-94774-052-4.
Le-Cam B, Luciana P, Arene L (2002). Evidence of two formae speciales in *Venturia inaequalis*, responsible for apple and pyracantha scab. *Phytopathol.* 92(3): 314-320.
Ma L, Zhang Z, Li J,Yang X, Fei B, Leung PHM, Tao XA (2019). New antimicrobial agent: Poly (3-hydroxybutyric acid) Oligomer. *Macromol. Biosci*. 5 (19). 2802-2815.
Maathuis FJM (2009). Physiological functions of mineral macronutrients*. Curr. Opinion in Plant Biol*. 12: 250-258.
Martin JT, Juniper BE (1970). The cuticles of plants. E. Arnolds Ltd., London. pp. 347.
Miroslavov EA (1994). Structural adaptations of plants to cold climate. *Bot. Zh*. 79(2): 20-26.
Miroslavov EA, Kravkin EM (1990). Comparative anatomy of the leaf of plants growing in mountains at different elevations. *Bot. Zh*. 75(3): 368-375.
Mizutani M, Kanaoka MM (2017). Environmental sensing and morphological plasticity in plants. Seminars in cell and developmental biology, New York, Academic 83: 69-77.
Morkunas I, Gmerek J (2007). The possible involvement of peroxidase in defense of yellow lupine embryo axes against Fusarium oxysporum. *J. Plant Physiol.* 164: 185-194.
Morkunas I, Ratajczak L (2014). The role of sugar signaling in plant defense responses against fungal pathogens. *Acta Physiol. Plant*. 36: 1607-1619.
Motyleva S. Upadysheva G, Tumaeva T (2021). The mineral composition of cherry (Prunus Cerasus Mill.) fruits depending on the scion-stock combination. *Potravinarstvo Slovak J. Food Sci*. 15: 74–82.
Motyleva SM (2018). The methodical recommendations on the implementation of ash elements and mineral inclusions analysis in plant organs by the method of energy dispersive spectrometry on an analytical REM - Moscow, FSBSI ARHIBAN; pp. 40. ISBN 978-5-00140-010-3.
Motyleva SM, Kuznetsov MN (2010). Micromorphology of leaves of triploid apple varieties immune to scab. *Sovrem. Sadovodstvo*. 1: 72-78.
Nieto-Penalver CG, Savino MJ, Bertini EV, Sanchez LA, de-Figueroa LIC (2014). Gluconic acid produced by Gluconacetobacter diazotrophicus Pal5 possesses antimicrobial properties. *Res. Microbiol.* 165: 549-558.
Pautov AA, Sapach YuO, Ivanova OV, Krylova EG (2014). Microrelief of the surface of leaves of flowering plants: stomatal rings and protrusions. *Bot. Zh.* 99(6): 625-640.
Pcries OS (1962). Studies on strawberry mildew, caused by Sphaerotheca macutaris (Wall, ex Fries) Jaczewski, II: Host parasite relationships on foliage of strawberry varieties. *Ann. Appl. Biol*. 50: 225-233.
Petkovsek MM, Slatnar A, Stampar F, Veberic R (2011). Phenolic compounds in apple leaves after infection with apple scab. *Biol. Plant*. 55(4): 725-730.
Potter D, Eriksson T, Evans RC, Oh S, Smedmark JEE, Morgan DR, Kerr M, Robertson KR, Arsenault M, Dickinson TA, Campbell CS (2007). Phylogeny and classification of Rosaceae. *Plant Sys. Evol*. 266: 5-43

Reddy VS, Reddy AS (2004). Proteomocs of calcium-signalling components in plants. *Phytochem*. 65: 1745-1776.

Riederer M, Muller C (2006). Annual plant reviews Biology of the plant cuticle. Wiley-Blackwell, pp.456.

Roberts FM, Martin JГ (1963). Withertip disease of limes (*Citrus aurantifolia*) in Zanzibar. Ill: The leaf cuticle in relation to infection by *Gloeosporium limettieola* Clausen. *Ann. Appl. Biol*. 51: 411-413.

Rolland F, Baena-Gonzalez E, Sheen J (2006). Sugar sensing and signaling in plants: conserved and novel mechanisms. *Annu. Rev. Plant Biol*. 57: 675-709.

Rosa M, Prado C, Podazza G, Interdonato R, Gonzalez JA, Hilal M (2009). Soluble sugars —metabolism, sensing and abiotic stress: a complex network in the life of plants. *Plant Signal Behav*. 4: 388-393.

Royer DL (2001). Stomatal density and stomatal index as indicators of paleoatmospheric CO2 concentration. *Rev. Palaeobot. Palynol*. 114: 1-28.

Sanders D, Pellow J, Brownlee C, Herper JF (2002). Calcium at the crossroads of signaling. *Plant Cell* 14: 101-417.

Savelyev NI (1998). The genetic basis of apple breeding. Michurunsk, pp, 304 (in Russian).

Savelyeva NN, Savelyeva IN (2012). Columnar apple tree (biology, genetics, breeding). Scientific publication, Michurinsk Research Center RF.-120s., il.-8s. ISBN 978-5-98429-093-7.

Sedov EN, Sedysheva GA, Zhdanov VV, Ulianovskaya EV, Serova ZM (2009). Breeding of Scab-resistant triploid apple varieties. *Vestnik VOGiS*. 13(4): 785-792 (in Russian).

Smolyakova VM, Yakuba GV (2003). Methodical in-structions on the diagnosis, accounting and forecasting of apple scab in the North Caucasus. Krasnodar: KRIA, 2003. pp. 44. (in Russian).

Takhtajan A (2009). Flowering plants. Springer.

Valsangiacomo C, Gessler C (1988). Role of the cuticular mem¬brane in ontogenic and Vf-resistance of apple leaves against *Venturia inaequalis*. *Phytopathol.* 78: 1066-1069.

Volk GM, Lynch-Holm VJ, Kostman TA, Goss LJ. Franceschi VR (2008). Plant biology First published (28): https://doi.org/10.1055/s-2002-20434.

Volonkov AS, Ivanova TV, Kumachova TK, Kozhevnikova AD, Tsydendambaev VD (2020). Polyunsaturated and very-long-chain acids are involved in the adaptation of maloideae (Rosaceae) to combinrd stress in the mountains. *Chem. Biodivers.* 17: 190588.

Ward D, Olsvig-Whittaker L (1993). Plant species diversity at the junction of two desert biogeographic zones. *Biodivers. Lett.* 1: 172-185.

White PJ (2004). Calcium signals in root cells: the roles of plasma membrane calcium channels. *Biologia* 59(S3): 77-83.

Wind J, Smeekens S, Hanson J (2010). Sucrose: metabolite and signaling molecule. *Phytochem*. 71: 1610-1614.

Yakuba GV (2013). Ecologized protection of apple scab in the face of climate change: monografiya. Krasnodar: GNU Severo-Kavkazskogo zonalnogo NII sadovodstva i vinogradarstva, pp. 213. (in Russian).

Yu S, Liang C, Liping Z, Diqiu Y (2010). Over expression of *OsWRKY72* gene interferes in the abscisic acid signal and auxin transport pathway of *Arabidopsis*. *J. Biosci*. 35: 459-471.

Zhdanov VV, Sedov EN (1991). Apple breeding for resistance to scab. Tula: Priok. kn. izd-vo, 1991. pp. 208. (in Russian).

Zhdanov VV, Zhuk GP (2006). Towards a problem of race specific apple resistance to scab. *Mikologiya and fitopatologiya*. 40(4): 314-319. (in Russian).
