# Supplementary material for: First assessment of Iranian pomegranate germplasm using targeted metabolites and morphological traits to develop the core collection and modeling of the current and future spatial distribution under climate change conditions
Source: PLoS One. 2023 Feb 3;18(2):e0265977. doi: 10.1371/journal.pone.0265977 (PMC9897574; doi:10.1371/journal.pone.0265977)
Supplement: Attachment — Submitted filename: response to reviewers.docx [file pone.0265977.s002.docx]

**Dear Dr. Mehdi Rahimi,**

We would like to thank you and the reviewers for constructive assessment of our manuscript entitled "**First assessment of Iranian pomegranate germplasm using targeted metabolites and morphological traits to develop the core collection and modeling of the current and future spatial distribution under climate change conditions**". We are pleased that all reviewers have found our article interesting. The authors greatly acknowledge the constructive comments made by the two reviewers. We have now revised the manuscript according to reviewers' comments, and a point-by-point response is submitted. Further, we confirmed that the funders had no role in study design, data collection and analysis, decision to publish, or preparation of the manuscript. Besides, the authors received no specific funding for this work.

We appreciate you for considering the revised version of the manuscript and look forward to receiving your decision.

Sincerely,

Mehrshad Zeinalabedini

*Mehrshad Zeinalabedini, PhD*

*Agricultural Biotechnology Research Institute of Iran (*[*www.abrii.ac.ir*](http://www.abrii.ac.ir/)*)*

*Mahdasht Road, Karaj, Iran. P.O. Box: 31535-1897*

*Tel: +98 (261) 2703536*

*Fax: +98 (261) 2704539*

*Email:* [mzeinolabedini@abrii.ac.ir](mailto:mzeinolabedini@abrii.ac.ir)

**Answers to editor comments**

Ref: PONE-D-22-06916

**Title:** First assessment of Iranian pomegranate germplasm using targeted metabolites and morphological traits to develop the core collection and modeling of the current and future spatial distribution under climate change conditions

**Journal:** Plos One

1. Please ensure that your manuscript meets PLOS ONE's style requirements, including those for file naming. The PLOS ONE style templates can be found at
   <https://journals.plos.org/plosone/s/file?id=wjVg/PLOSOne_formatting_sample_main_body.pdf> and 
   <https://journals.plos.org/plosone/s/file?id=ba62/PLOSOne_formatting_sample_title_authors_affiliations.pdf>

Re: The format was checked and adjusted based on the editorial comments.

2. We note that the grant information you provided in the ‘Funding Information’ and ‘Financial Disclosure’ sections do not match.

When you resubmit, please ensure that you provide the correct grant numbers for the awards you received for your study in the ‘Funding Information’ section.

Re: This project was supported by the Agricultural Biotechnology Research Institute of Iran, ABRII07-05-05-92117. We revised this part based on the reviewer comment.

3. Thank you for stating the following financial disclosure:

The funders had no role in study design, data collection and analysis, decision to publish, or preparation of the manuscript. At this time, please address the following queries:

Please clarify the sources of funding (financial or material support) for your study. List the grants or organizations that supported your study, including funding received from your institution. State what role the funders took in the study. If the funders had no role in your study, please state: "The funders had no role in study design, data collection and analysis, decision to publish, or preparation of the manuscript." If any authors received a salary from any of your funders, please state which authors and which funders. If you did not receive any funding for this study, please state: "The authors received no specific funding for this work." Please include your amended statements within your cover letter; we will change the online submission form on your behalf.

Re: we amended the requested statements in the cover letter based on the editorial comments.

4. Thank you for stating the following in your Competing Interests section:

I have read the journal's policy and the authors of this manuscript have the following competing interests
 
Please complete your Competing Interests on the online submission form to state any Competing Interests. If you have no competing interests, please state "The authors have declared that no competing interests exist.", as detailed online in our guide for authors at <http://journals.plos.org/plosone/s/submit-now> 
 This information should be included in your cover letter; we will change the online submission form on your behalf.

Re: we amended the requested statements based on the editorial comments.

5. In your Data Availability statement, you have not specified where the minimal data set underlying the results described in your manuscript can be found. PLOS defines a study's minimal data set as the underlying data used to reach the conclusions drawn in the manuscript and any additional data required to replicate the reported study findings in their entirety. All PLOS journals require that the minimal data set be made fully available. For more information about our data policy, please see <http://journals.plos.org/plosone/s/data-availability>.

Upon re-submitting your revised manuscript, please upload your study’s minimal underlying data set as either Supporting Information files or to a stable, public repository and include the relevant URLs, DOIs, or accession numbers within your revised cover letter. For a list of acceptable repositories, please see <http://journals.plos.org/plosone/s/data-availability#loc-recommended-repositories>. Any potentially identifying patient information must be fully anonymized.

Important: If there are ethical or legal restrictions to sharing your data publicly, please explain these restrictions in detail. Please see our guidelines for more information on what we consider unacceptable restrictions to publicly sharing data: <http://journals.plos.org/plosone/s/data-availability#loc-unacceptable-data-access-restrictions>. Note that it is not acceptable for the authors to be the sole named individuals responsible for ensuring data access.

We will update your Data Availability statement to reflect the information you provide in your cover letter.

Re: The data set used in this study was presented in Supplementary Table 1 (S1 Table).

6. PLOS requires an ORCID iD for the corresponding author in Editorial Manager on papers submitted after December 6th, 2016. Please ensure that you have an ORCID iD and that it is validated in Editorial Manager. To do this, go to ‘Update my Information’ (in the upper left-hand corner of the main menu), and click on the Fetch/Validate link next to the ORCID field. This will take you to the ORCID site and allow you to create a new iD or authenticate a pre-existing iD in Editorial Manager. Please see the following video for instructions on linking an ORCID iD to your Editorial Manager account: <https://www.youtube.com/watch?v=_xcclfuvtxQ>

Re: ORCID iD for *Mehrshad Zeinalabedini as* the corresponding author is 0000-0002-34364334.

7. We note that Figures 1-3 and 13-14 in your submission contain [map/satellite] images which may be copyrighted. All PLOS content is published under the Creative Commons Attribution License (CC BY 4.0), which means that the manuscript, images, and Supporting Information files will be freely available online, and any third party is permitted to access, download, copy, distribute, and use these materials in any way, even commercially, with proper attribution. For these reasons, we cannot publish previously copyrighted maps or satellite images created using proprietary data, such as Google software (Google Maps, Street View, and Earth). For more information, see our copyright guidelines: <http://journals.plos.org/plosone/s/licenses-and-copyright>.
 We require you to either (1) present written permission from the copyright holder to publish these figures specifically under the CC BY 4.0 license, or (2) remove the figures from your submission:
 a. You may seek permission from the original copyright holder of Figures 1-3 and 13-14 to publish the content specifically under the CC BY 4.0 license.  
 We recommend that you contact the original copyright holder with the Content Permission Form (<http://journals.plos.org/plosone/s/file?id=7c09/content-permission-form.pdf>) and the following text:
“I request permission for the open-access journal PLOS ONE to publish XXX under the Creative Commons Attribution License (CCAL) CC BY 4.0 (<http://creativecommons.org/licenses/by/4.0/>). Please be aware that this license allows unrestricted use and distribution, even commercially, by third parties. Please reply and provide explicit written permission to publish XXX under a CC BY license and complete the attached form.”
Please upload the completed Content Permission Form or other proof of granted permissions as an "Other" file with your submission.
 
In the figure caption of the copyrighted figure, please include the following text: “Reprinted from [ref] under a CC BY license, with permission from [name of publisher], original copyright [original copyright year].”
 b. If you are unable to obtain permission from the original copyright holder to publish these figures under the CC BY 4.0 license or if the copyright holder’s requirements are incompatible with the CC BY 4.0 license, please either i) remove the figure or ii) supply a replacement figure that complies with the CC BY 4.0 license. Please check copyright information on all replacement figures and update the figure caption with source information. If applicable, please specify in the figure caption text when a figure is similar but not identical to the original image and is therefore for illustrative purposes only.

Re: Figures 1-3 and 13-14 were designed by the free software, Diva-GIS (available at: http://www.diva-gis.org/climate; accessed on 4th October 2004) and MAXENT (<https://biodiversityinformatics.amnh.org/open_source/maxent/>) to construct the maps by the authors and had no copyright.

**Answers to Reviewers comments**

Reviewer #1

1. What is novelty in the abstract? It needs improvement.

Re: Pomegranate core collection was established for the first time in Iran by combining morphological and metabolic data. Also, the impact of future climatic conditions (until the middle of the century) on the geographical distribution of pomegranate had not been investigated to date, which was predicted in this study. This information will help us identify the most suitable places for orchard construction in the future.

2. Reference should be according to journal format

Re: References were formatted according to the NLM/ICMJE style (the journal format).

4. Line 302 “The analysis of Spearman coeﬃcient of correlation between morphological and metabolite traits was showed antioxidant activity correlated positively with total phenol”. Rephrase sentence.

Re: it was revised.

5. Line 312. “also reported there was a negative correlation”. Rephrase the sentence..

Re: The sentence was rephrased.

5. Do add the following latest references in discussion part for justification of your results, please.

Zarei A (2017). Biochemical and pomological characterization of pomegranate accessions in fars province of Iran. SABRAO J. Breed. Genet. 49(2): 155–167.

Tarinta T, Chanthai S, Lertrat K, Nawata E, Techawongstien S (2020). Identification of the secondary metabolite capsiate in Capsicum germplasm accessions. SABRAO J. Breed. Genet. 52(2):144–157.

Upadyshev MT (2022). Apple cultivars and rootstocks assay for the identification of diverse viruses and healthy genotypes for breeding. SABRAO J. Breed. Genet. 54(1): 79-87. <http://doi.org/10.54910/sabrao2022.54.1.8>.

Motyleva SM, Medvedev SM, Morozova NG, Kulikov IM (2021). Leaf micromorphological and biochemical features of scab disease in immune and moderately resistant columnar apple (Malus domestica) cultivars. SABRAO J. Breed. Genet. 53(3): 352-366.

Hong-Qun, L. I., Li-Gang, X. I. N. G., & Xie-Ping, S. U. N. (2022). Predicting the potential distribution of Taxus wallichiana var. Mairei under climate change in China using maxent modeling. Pak. J. Bot. 54(4): 1305-1310.

Ahmad, N., Shakil, A., Shinwari, Z. K., Ahmad, I., & Wahab, A. (2022). Phytochemical study and antimicrobial activities of extracts and its derived fractions obtained from Berberis vulgaris L. and Stellaria media L. leaves. Pak. J. Bot. 54(4): 1517-1521.

Re: Some of these articles were added in the results and discussion section, but we did not have access to the full article of some of them. As a result, we were unable to study them and add them to our article.

Reviewer #2:

- In Materials & Methods, authors explained that 152 genotypes selected from 760 genotypes based on the results of Kazemi alamuti et al. [26]. It is better to explain in brief how and why these genotypes selected and present the genotypes name, source/origin (if applicable) as a supplementary Table. I would appreciate to see official reference numbers for all accessions so that they can be order from reference gene banks and benefit for the entire pomegranate scientists community.

Re: The genotypes in this study were selected based on the previous results of our different studies (Kazemi alamuti et al. 2012; Mousavi Derazmahalleh et al. 2013; Razi et al. 2021), which were performed on various objects e.g. genetic diversity, population genetic etc. of Yazd pomegranate national germplasm. The name and origin of genotypes also was presented in S1 Table based on the existing codes of Iranian pomegranate germplasm.

- In Materials & Methods, section “Investigation of morphological characteristics”: explain how many plants per genotypes evaluated? Some information about the field? Are there any statistical design? Or just sampled from a tree in a collection site? Please clarify it.

Re: The number of plants for evaluating each genotype was three individual from which eight samples were harvested.

In this collection, four trees per genotype have been cultivated in five blocks in an area of 19 hectares. Information on the geographical location and climatic conditions of the site of collection was mentioned in the article.

- Line 124, page 6: “The pH measurement was performed using a digital pH meter (Metrohm model) at 21 °C”, pH of what? Clearly explain.

Re: pH of pomegranate aril juice was measured.

- Fig 4. Spearman correlation matrix of the traits. This figure is difficult for reader to well understanding the relationship between traits. It is recommended to summarized in a Table.

Re: Another figure, which includes the correlation coefficient values, replaced this figure. In the new figure, determining the correlation through both the values of the correlation coefficient and the color spectrum will make it easier for the reader to understand the correlation between traits.

- I strongly recommended to present a Table about some statistical parameters for all measured traits, like as mean, maximum, minimum, standard deviation and coefficient of variation (CV). This can be better explain the amount of variation in the studied germplasm.

Re: Descriptive statistics of biochemical traits (minimum, maximum, mean, and coefficient of variation) of 152 pomegranate genotypes were presented in Table 2.

The morphological features studied were qualitative traits that were scored and evaluated.

- Are there any ANOVA analysis for traits?

Re: there was no ANOVA analysis for traits in this study.
