# Supplementary material for: First assessment of Iranian pomegranate germplasm using targeted metabolites and morphological traits to develop the core collection and modeling of the current and future spatial distribution under climate change conditions
Source: PLoS One. 2023 Feb 3;18(2):e0265977. doi: 10.1371/journal.pone.0265977 (PMC9897574; doi:10.1371/journal.pone.0265977)
Supplement: Attachment — Submitted filename: PONE-22-06916 - Review Report - May 31, 2022.doc [file pone.0265977.s003.doc]

Manuscript titled:

# First assessment of Iranian pomegranate germplasm using targeted metabolites and morphological traits to develop the core collection and modeling of the current and future spatial distribution under climate change conditions

There are several linguistic and typographical mistakes in this paper that must be corrected.

1. What is novelty in the abstract? It needs improvement
2. Reference should be according to journal format
3. The manuscript must revise with native English speakers because the manuscript contains a lot of mistakes in grammar and structure, for example, Line 302

“The analysis of Spearman coeﬃcient of correlation between morphological and metabolite traits **was s**howed antioxidant activity correlated positively with total phenol”. Rephrase sentence.

4. Line 312. “also reported there was a negative correlation”. Rephrase the sentence.
5. The discussion section is just descriptive of the results, there are no logical reasoning. Give some recent and comprehensive literature in discussion and support the obtained results. Do not give simple biological inference.
6. Do add the following latest references in discussion part for justification of your results, please.

Zarei A (2017). [Biochemical and pomological characterization of pomegranate accessions in fars province of Iran](http://bit.ly/Zarei-49-2-June2017). SABRAO J. Breed. Genet. 49(2): 155–167.

Tarinta T, Chanthai S, Lertrat K, Nawata E, Techawongstien S (2020). [Identification of the secondary metabolite capsiate in Capsicum germplasm accessions](http://sabraojournal.org/identification-of-the-secondary-metabolite-capsiate-in-capsicum-germplasm-accessions/). SABRAO J. Breed. Genet. 52(2):144–157.

Upadyshev MT (2022). [Apple cultivars and rootstocks assay for the identification of diverse viruses and healthy genotypes for breeding.](https://sabraojournal.org/apple-cultivars-and-rootstocks-assay-for-the-identification-of-diverse-viruses-and-healthy-genotypes-for-breeding/) SABRAO J. Breed. Genet. 54(1): 79-87. http://doi.org/10.54910/sabrao2022.54.1.8.

Motyleva SM, Medvedev SM, Morozova NG, Kulikov IM (2021). [Leaf micromorphological and biochemical features of scab disease in immune and moderately resistant columnar apple (*Malus domestica*) cultivars](https://sabraojournal.org/wp-content/uploads/2021/09/SABRAO-J-Breed-Genet-533-352-366-MOTYLEVA.pdf). SABRAO J. Breed. Genet. 53(3): 352-366.

Hong-Qun, L. I., Li-Gang, X. I. N. G., & Xie-Ping, S. U. N. (2022). Predicting the potential distribution of *Taxus wallichiana* var. Mairei under climate change in China using maxent modeling. Pak. J. Bot. 54(4): 1305-1310.

Ahmad, N., Shakil, A., Shinwari, Z. K., Ahmad, I., & Wahab, A. (2022). Phytochemical study and antimicrobial activities of extracts and its derived fractions obtained from *Berberis vulgaris* L. and *Stellaria media* L. leaves. Pak. J. Bot. 54(4): 1517-1521.

Regards
