# Supplementary material for: First assessment of Iranian pomegranate germplasm using targeted metabolites and morphological traits to develop the core collection and modeling of the current and future spatial distribution under climate change conditions
Source: PLoS One. 2023 Feb 3;18(2):e0265977. doi: 10.1371/journal.pone.0265977 (PMC9897574; doi:10.1371/journal.pone.0265977)
Supplement: Attachment — Submitted filename: Response to Reviewers.docx [file pone.0265977.s004.docx]

Dear Dr. Mehdi Rahimi,

Dear Dr. Mehdi Rahimi,

Thank you for giving me the opportunity to submit a revised draft of my manuscript titled "**First assessment of Iranian pomegranate germplasm using targeted metabolites and morphological traits to develop the core collection and modeling of the current and future spatial distribution under climate change conditions**”. I appreciate the time and effort that you and the reviewers have dedicated to providing your valuable feedback on our manuscript. I hope the manuscript after careful revisions meet PLOS ONE’s publication criteria.

I appreciate you for considering the revised version of the manuscript and look forward to receiving your decision.

Sincerely,

Mehrshad Zeinalabedini

*Mehrshad Zeinalabedini, PhD*

*Agricultural Biotechnology Research Institute of Iran (*[*www.abrii.ac.ir*](http://www.abrii.ac.ir/)*)*

*Mahdasht Road, Karaj, Iran. P.O. Box: 31535-1897*

*Tel: +98 (261) 2703536*

*Fax: +98 (261) 2704539*

*Email:* [mzeinolabedini@abrii.ac.ir](mailto:mzeinolabedini@abrii.ac.ir)

Here is a response to the reviewers’ comment.

Reviewer #1: A Confusion:
It’s not clear that the Authors incorporated or not the below suggested References, and 2/3 files of the Article are merged, and it not also clear that which one is revised version, please.
6. Do add the following latest references in discussion part for justification of your results, please.
Zarei A (2017). Biochemical and pomological characterization of pomegranate accessions in fars province of Iran. SABRAO J. Breed. Genet. 49(2): 155–167.
Tarinta T, Chanthai S, Lertrat K, Nawata E, Techawongstien S (2020). Identification of the secondary metabolite capsiate in Capsicum germplasm accessions. SABRAO J. Breed. Genet. 52(2):144–157.
Upadyshev MT (2022). Apple cultivars and rootstocks assay for the identification of diverse viruses and healthy genotypes for breeding. SABRAO J. Breed. Genet. 54(1): 79-87.

Motyleva SM, Medvedev SM, Morozova NG, Kulikov IM (2021). Leaf micromorphological and biochemical features of scab disease in immune and moderately resistant columnar apple (Malus domestica) cultivars. SABRAO J. Breed. Genet. 53(3): 352-366.
Hong-Qun, L. I., Li-Gang, X. I. N. G., & Xie-Ping, S. U. N. (2022). Predicting the potential distribution of Taxus wallichiana var. Mairei under climate change in China using maxent modeling. Pak. J. Bot. 54(4): 1305-1310.
Ahmad, N., Shakil, A., Shinwari, Z. K., Ahmad, I., & Wahab, A. (2022). Phytochemical study and antimicrobial activities of extracts and its derived fractions obtained from Berberis vulgaris L. and Stellaria media L. leaves. Pak. J. Bot. 54(4): 1517-1521.

Re: Some of these articles (Zarei 2017; Hong-Qun et al. 2022) had been added in the results and discussion section, and I highlighted them again in the manuscript. We did not have access to the full article of Motyleva et al. (2021). As a result, we were unable to study it and add it to our article. The rest of the mentioned references could not be added to the article due to their completely different topic from the objective of this study.
