# Supplementary material for: First assessment of Iranian pomegranate germplasm using targeted metabolites and morphological traits to develop the core collection and modeling of the current and future spatial distribution under climate change conditions
Source: PLoS One. 2023 Feb 3;18(2):e0265977. doi: 10.1371/journal.pone.0265977 (PMC9897574; doi:10.1371/journal.pone.0265977)
Supplement: Attachment — Submitted filename: response to reviewers.docx [file pone.0265977.s006.docx]

Dear Richard Ibañez Dilla

Many thanks for your email informing us to revise our manuscript.

We addressed the following issues.

1. If possible, please upload an updated file showing your changes either highlighted or using track changes.  This should be uploaded as a Revised Manuscript w/tracked changes, file type. Please follow this link for more information: [http://blogs.PLOS.org/everyone/2011/05/10/how-to-submit-your-revised-manuscript/](http://blogs.plos.org/everyone/2011/05/10/how-to-submit-your-revised-manuscript/)

Re. In response to this issue, it is necessary to explain that in the file "Response to reviewers" that we sent the last time, we stated that since there was no change in the manuscript, we uploaded two files 'Response to Reviewers' and 'Manuscript'.

We upload new file "revised manuscript with track changes".

2. Please amend the manuscript submission data (via Edit Submission) to include all the authors.

Re: we amended the manuscript submission data to include all the authors

3. Please amend your list of authors on the manuscript to ensure that each author is linked to an affiliation.

Re: we amended our list of authors on the manuscript so that each author is linked to an affiliation.

We hope that we have satisfactory tackled all issues raised and that the manuscript is now well suited for publication.

We appreciate you for considering the revised version of the manuscript and look forward to receiving your decision.

Sincerely,

Mehrshad Zeinalabedini
